# *Neurospora crassa* NADPH Oxidase NOX-1 Is Localized in the Vacuolar System and the Plasma Membrane

**DOI:** 10.3389/fmicb.2019.01825

**Published:** 2019-08-14

**Authors:** Nallely Cano-Domínguez, Barry Bowman, Leonardo Peraza-Reyes, Jesús Aguirre

**Affiliations:** ^1^Departamento de Biología Celular y del Desarrollo, Instituto de Fisiología Celular, Universidad Nacional Autónoma de México, Mexico City, Mexico; ^2^Department of Molecular, Cell and Developmental Biology, University of California, Santa Cruz, Santa Cruz, CA, United States; ^3^Departamento de Bioquímica y Biología Estructural, Instituto de Fisiología Celular, Universidad Nacional Autónoma de México, Mexico City, Mexico

**Keywords:** NOR-1, ROS, fungal development, cell fusion, fungal growth, CAT fusion

## Abstract

The NADPH oxidases (NOX) catalyze the production of superoxide by transferring electrons from NADPH to O_2_, in a regulated manner. In *Neurospora crassa* NOX-1 is required for normal growth of hyphae, development of aerial mycelium and asexual spores, and it is essential for sexual differentiation and cell-cell fusion. Determining the subcellular localization of NOX-1 is a critical step in understanding the mechanisms by which this enzyme can regulate all these different processes. Using fully functional versions of NOX-1 tagged with mCherry, we show that in growing hyphae NOX-1 shows only a minor association with the endoplasmic reticulum (ER) markers Ca^2+^-ATPase NCA-1 and an ER lumen-targeted GFP. Likewise, NOX-1 shows minor co-localization with early endosomes labeled with YPT-52, a GTPase of the Rab5 family. In contrast, NOX-1 shows extensive co-localization with two independent markers of the entire vacuolar system; the vacuolar ATPase subunit VMA-1 and the fluorescent molecule carboxy-DFFDA. In addition, part of NOX-1 was detected at the plasma membrane. The NOX-1 regulatory subunit NOR-1 displays a very different pattern of localization, showing a fine granular distribution along the entire hypha and some accumulation at the hyphal tip. In older hyphal regions, germinating conidia, and conidiophores it forms larger and discrete puncta some of which appear localized at the plasma membrane and septa. Notably, co-localization of NOX-1 and NOR-1 was mainly observed under conidial cell-cell fusion conditions in discrete vesicular structures. NOX functions in fungi have been evaluated mainly in mutants that completely lacked this protein, also eliminating interactions between hyphal growth regulatory proteins NOR-1, the GTPase RAC-1 and the scaffold protein BEM-1. To dissect NOX-1 roles as scaffold and as ROS-producing enzyme, we analyzed the function of NOX-1::mCherry proteins carrying proline 382 by histidine (P382H) or cysteine 524 by arginine (C524R) substitutions, predicted to only affect NADPH-binding. Without notably affecting NOX-1 localization or protein levels, each of these substitutions resulted in lack of function phenotypes, indicating that NOX-1 multiple functions are all dependent on its oxidase activity. Our results open new interpretations to possible NOX functions, as components of the fungal vacuolar system and the plasma membrane, as well as to new vacuolar functions.

## Introduction

Many years ago we proposed reactive oxygen species (ROS) as signaling molecules and regulators of cell differentiation (Hansberg and Aguirre, 1990; Aguirre et al., 2005). The existence of NADPH oxidase (NOX) enzymes, capable of producing ROS in a highly regulated fashion, became particularly interesting to us in this context. NOX2 (gp91*phox*), the catalytic component of the first and most studied NOX was reported as a novel cytochrome *b*558 in phagocytic vacuoles of human granulocytes (Segal and Jones, 1978), where phagocytosis is associated with the production of superoxide and other derived ROS, necessary for the efficient killing of invading pathogens. Defects in NOX components cause the immunodeficiency syndrome called granulomatous disease or CGD (Babior, 1995). Later, it was found that NOX2 was part of a larger family of enzymes that were ubiquitous in eukaryotic cells, where they were related not only to innate immunity but also to other processes such as modifications of the extracellular matrix, cell-cell signaling, development, and morphogenesis (Lambeth et al., 2000; Lara-Ortiz, 2004; Aguirre and Lambeth, 2010).

We reported the presence of three different NOX subfamilies in fungi and the Amoebozoa, and demonstrated that NOX enzymes were essential for ROS production and sexual development in the Ascomycetes *Aspergillus nidulans* (Lara-Ortiz et al., 2003; Aguirre et al., 2005), *Neurospora crassa* (Cano-Dominguez et al., 2008), and *Sordaria macrospora* (Dirschnabel et al., 2014). Independently, Malagnac et al. (2004) found that in *Podospora anserina* PaNox1 and PaNox2 were needed for sexual development and ascospore germination, respectively (Malagnac et al., 2004). Since then, fungal NOX enzymes have been linked to other critical functions such as growth, asexual development, cell-cell fusion (Cano-Dominguez et al., 2008; Fu et al., 2011; Read et al., 2012; Roca et al., 2012; Dirschnabel et al., 2014), pathogenicity (Egan et al., 2007; Segmuller et al., 2008; Kim et al., 2011; Schurmann et al., 2013; Yang and Chung, 2013; Zhang et al., 2016; Rossi et al., 2017; Zhou et al., 2017), endosymbiosis (Tanaka et al., 2006), and the asexual reproduction induced by injury in *Trichoderma atroviride* (Hernandez-Onate et al., 2012). More recently the functions of other NOX enzymes, originally considered as ferric reductases, have been reported in *Saccharomyces cerevisiae* (Rinnerthaler et al., 2012) and *Candida albicans* (Rossi et al., 2017), while bioinformatic analysis and *in vitro* experiments suggest the presence of NOX enzymes in prokaryotes (Hajjar et al., 2017). Such ubiquity of NOX enzymes supports the idea that the controlled production of ROS as signaling molecules appeared early in life evolution as an important mechanism to regulate cell physiology and differentiation.

In mammalian phagocytic cells part of NOX2 is localized in the plasma membrane, where it interacts with subunits p22*phox*, p40*phox*, p47*phox*, p67*phox*, and the GTPase Rac1 for its activation [reviewed in Aguirre and Lambeth (2010)]. Fungal A and B NOX subfamilies belong to the NOX2 family (Lara-Ortiz et al., 2003; Aguirre et al., 2005), and interact with equivalent subunits. Indeed, p67*phox* homolog NoxR interacts with RacA (Takemoto et al., 2006), and together with p22*phox* homolog NoxD (Lacaze et al., 2015; Siegmund et al., 2015) are required for NOX functions (Takemoto et al., 2006; Cano-Dominguez et al., 2008). Membrane proteins of the tetraspanin family have also been linked to NOX function (Lambou et al., 2008; Schurmann et al., 2013; Siegmund et al., 2013; Lacaze et al., 2015; Zhao et al., 2016) and suggested to play roles analogous to p22*phox* for NoxB family members. Finally, it has been proposed that fungal Bem1 proteins play roles analogous to animal p40*phox* and p47*phox* (Takemoto et al., 2011).

Although little is known about how NOX and ROS can regulate so many seemingly different functions in fungi, some NOX functions have been related to actin organization and calcium signaling. In yeast, mutants lacking the NOX Yno1p are hypersensitive to drugs that inhibit F-actin nucleation and elongation, and this phenotype is rescued by non-toxic concentrations of H_2_O_2_, suggesting that Yno1p is necessary for proper F-actin function (Rinnerthaler et al., 2012). In the phytopathogen *Magnaporthe grisea* *Δnox1* and *Δnox2* mutants are able to differentiate the leaf penetration structure called appressorium, but fail to penetrate the plant cuticle (Egan et al., 2007). Nox2 and NoxR are required to organize a septin ring at the base of the appressorium pore, which in turn is necessary for the assembly of a toroidal F-actin network at the point of penetration peg emergence, while Nox1 is needed to maintain the cortical F-actin network (Ryder et al., 2013). A similar situation occurs in the fungus *Verticillium dahlia*, which only infects plant roots using a structure called hyphopodium, from which a penetration peg develops. Mutants lacking NOX VdNoxB are impaired in the formation of a septin ring that partitions the hyphopodium and the invasive hyphae, which is critical for the secretion of effector proteins (Zhou et al., 2017). Moreover, tetraspanin VdPls1 and NOX VdNoxB are specifically expressed in hyphopodia, where they colocalize and are necessary for ROS production and the development of the penetration peg. The nuclear targeting of the calcium-regulated transcription factor VdCrz1, which normally occurs in hyphopodia, was prevented in mutants lacking VdPls1 or VdNoxB, linking NOX function and calcium signaling in this process (Zhao et al., 2016).

*Neurospora crassa* is a particularly interesting model to study NOX function, as it contains two NOX enzymes (NOX-1 and NOX-2), both requiring the regulatory subunit encoded by *nor-1* (NCU07850). While *nox-2* (NCU10775) elimination only affects ascospore germination, *nox-1* (NCU02110) deletion has more pleitropic effects in this fungus than in any other fungus studied. Indeed, NOX-1 is required for normal growth of vegetative hyphae, growth of the aerial mycelium and conidia production, and it is essential for sexual differentiation and hyphal fusion (Cano-Dominguez et al., 2008; Read et al., 2012). To understand how NOX-1 might regulate so many different cellular processes, it is critical to know the subcellular localization of this enzyme. Here we compare the subcellular localization of NOX-1 and its regulatory subunit NOR-1 during different conditions of growth and development and provide genetic evidence supporting that all critical NOX-1 functions require its oxidase activity.

## Materials and Methods

### Strains, Media, Growth Conditions, and Phenotypic Characterization

*Neurospora crassa* strains used in this work are listed in Table S1. All strains were grown on Vogel's minimal medium (VMM) solidified with 1.8% agar at 30°C (Davies and deSerres, 1970). For histidine auxotrophic strains, VMM was supplemented with 0.002% of L-histidine. To determine the production of conidia, 1 × 10^4^ conidia were inoculated on borosilicate tubes containing 3 mL of solid VMM and incubated at 30°C during 3 days in the dark plus 2 days at 25°C in the light. Conidia from these tube cultures were collected with 1 mL of sterile water, diluted and counted using a Neubauer chamber. For radial growth analysis, 1 × 10^3^ conidia were inoculated on the center of a Petri dish containing Vogel's solid minimal medium. Radial growth was measure after 24 h at 30°C and plates were photographed. For observation of conidial anastomosis tube (CAT) formation and fusion, 8 × 10^6^ conidia were inoculated on solid MMV medium using a glass handle, and incubated at 37°C for 4–5 h. An agar block sectioned from these cultures was directly observed under the microscope. To induce sexual crosses, conidia were inoculated on synthetic crossing medium and incubated for 6 days at 25°C in the light (Westergaard and Mitchell, 1974). After that time, a conidial suspension from the opposite mating type was laid over this culture. To follow fruiting body primordia (protoperithecia) development, 1 × 10^3^ conidia were used to initiate cultures according to the method of Bistis (Bistis, 1983; Cano-Dominguez et al., 2008).

### Plasmids Construction

The list of plasmids obtained in this work is given in Table S2. To construct plasmid pNCNOX-1::mCherry4, *nox-1* (NCU02110) ORF (1659 pb) was amplified using *N. crassa* genomic DNA as template and primers NOX-1 XbaIF2 and NOX-1 PacIR2 (Table S3). The generated PCR product fragment was cloned as a *Xba*I/*Pac*I fragment into plasmid pJV15-2 (Verdin et al., 2009). Plasmids pNCNOX-1P382H and pNCNOX-1C524R were generated using In-Fusion® HD Cloning Kit-Clonetech and primers Nox-1 P382HF and Nox-1 P382HR for pNCNOX-1P382H, and Nox-1 C524RF and Nox-1 C524RR for pNCNOX-1C524R. These primers contain point mutations and 15 pb overlap with adjoining fragments. Plasmid pNCNOX-1::mCherry4 was used as template for the In-Fusion® reaction. To replace *ccg-1* promoter by *nox-1* native promoter in pNCNOX-1::mCherry4, we used genomic DNA to amplify a 1226 pb upstream of *nox-1* ORF with primes NOX-1PROM NATF and PromNatR. Then, we amplified the host plasmid pNCNOX-1::mCherry4 using primers PNC-NOX-1mCherry PromNatF and PNC-NOX-1mCherry PromNatR. The amplified host plasmid lacks the *ccg-1* promoter and contains 15 pb overlap with the 1226 pb *nox-1* promoter region. In the last step, we used In-Fusion® HD Cloning Kit-Clonetech to perform the ligation. To fuse NOR-1 to GFP, we amplified *nor-1* (NCU07850) ORF as a 1893 pb fragment, using primers NOR-1BAMHIUP and NOR-1PacILOW and genomic DNA as template. This PCR product was purified and cloned into pGEM®-T Easy Vector (Promega, Madison, WI) to yield plasmid pGemNOR-1-2. A *nor-1 PacI-BamHI* fragment obtained from pGemNOR-1-2 was cloned into plasmid pMF272 (Freitag et al., 2004), digested with *XbaI/PacI*, to generate pNCNOR-1::GFP18.

### *Neurospora crassa* Transformation

Conidia were collected from VMM slant cultures using cold sterile water. After determining their concentration, 1.25 × 10^8^ conidia were collected by centrifugation and washed two times with 500 μL of 1 M cold sorbitol and finally resuspended in 40 μL of the same solution. This spore suspension was incubated with DNA (plasmids or PCR products, during 30 min at 4°C) and electroporated using the Gene Pulser II and Pulser Controller II (Bio-Rad, Hercules, CA) set to 1.5 kV, 600 Ω, and 2.5 μF, as reported (Colot et al., 2006). After electroporation, 960 μL of 1 M cold sorbitol were added, mixed with 25 mL of recovery solution (liquid Vogel's medium) and incubated at 30°C and 100 RPM, for 2 h. This solution was mixed with 25 mL of regeneration agar (Vogel's medium with 1 M sorbitol, 2% L-sorbose, 0.05% glucose, 0.05% fructose, and 1% agar) and plated immediately on solid CA medium (2% L-sorbose, 0.05% glucose, 0.05% fructose in Vogel media, and 1% agar) containing hygromycin (200 μg/mL) or phleomycin (80 μg/mL) (Colot et al., 2006). Transformants were purified to obtain homokaryotic strains through three monosporic passes on CA medium plus hygromycin (200 μg/mL) or phleomycin (80 μg/mL).

### Heterokaryon Formation

To observe hypha with two proteins labeled with different fluorophores, we constructed heterokaryons using strains with the same mating types. We inoculated 4 × 10^6^ conidia of each strain on a solid VMM. Cultures were incubated at 37°C for 4–5 h to induce CAT fusion. Cell fusion was verified under epifluorescence microscopy and then agar blocks were transferred to solid VMM and incubated for 12 h at 30°C. The margins of the colony were used to detect hyphae expressing both markers and observed under confocal microscopy.

### Microscopy

Primordia (protoperithecia) and mature sexual fruiting bodies (perithecia) were photographed using a Nikon stereoscopic microscope. Live-cell imaging was carried out using the “inverted agar block” method (Hickey et al., 2002) and a Zeiss LSM-800-Inverted Laser Scanning Confocal Microscope with a Plan-Apochromatic 63x/1.4 oil immersion objective and 488 and 561 nm laser lines, or a Zeiss LSM-800-Inverted Laser Scanning Confocal Microscope with a 30°C temperature chamber and the same parameters. Time-lapse images were recorded simultaneously by bright field microscopy and fluorescence confocal microscopy. The images were processed using ZEN 2012 (Carl Zeiss, Jena, Germany) software. Latrunculin A (Lat A) (Tocris Bioscience, Bristol, UK: 100 μg) was use to inhibit actin polymerization and endocytosis. A 2 μg/μL stock solution in DMSO was diluted with VMM to a final 20 μg/mL working concentration. Fifty microliter of this solution were placed onto a coverslip and mycelium on an agar block was placed in contact with the latrunculin. After 10 min, the sample was observed using confocal microscopy. Oregon Green 488 carboxylic acid diacetate (carboxy-DFFDA; Molecular Probes) was used to visualize the vacuolar system. A 1 mM stock solution in DMSO was diluted with VMM to a final 10 μM concentration. Fifty microliter of this solution were placed onto a coverslip and conidia or mycelium on agar blocks were placed in contact with the dye. The sample was observed immediately in the Zeiss LSM-800 Confocal Microscope equipped with a 30°C temperature chamber.

## Results

### NOX-1::mCherry Expressed From *ccg*-1 Promoter Fully Complements *Δnox-1* Mutant Multiple Defects

To determine NOX-1 subcellular localization we decided to use fluorescent protein mCherry, an improved version of mRFP1, showing higher photostability and pH resistance (Shaner et al., 2004). First, we generated plasmid pNCNOX-1::mCherry4, which contains a *nox-1::mCherry* gene fusion under the control the *ccg-1* constitutive promoter and allows integration at the *his-3* locus. We used this plasmid to transform strain *Δnox-1 his-3*, and four His-3^+^ transformants were analyzed by PCR to confirm the presence of this plasmid at the *his-3* locus. Strains *Δnox-1 nox-1::mCherry d* and *e* were selected for further experiments (Figure S1). As reported before (Cano-Dominguez et al., 2008), *Δnox-1* mutants showed a severe decrease in aerial mycelium growth and conidiation, reduced radial growth and were unable to develop perithecia, with these phenotypes being somewhat enhanced by the presence of *his-3* mutation (Figures S2A–E). The *nox-1::mCherry* allele was able to fully complement the defects in aerial mycelium growth and conidiation (Figures S2A,B) and restored radial growth (Figures S2C,D, S7D). Moreover, *Δnox-1* complemented strains were able to produce protoperithecia (not shown) that were competent for fertilization and able to develop mature perithecia (Figure S2E), containing viable ascospores (not shown).

**Figure 1:**
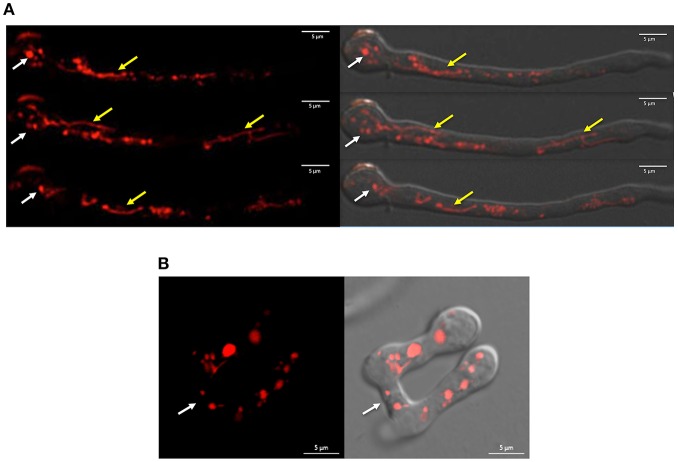
NOX-1::mCherry expressed from *ccg*-1 promoter is localized in discrete compartments and complements *Δnox-1* cell fusion defects. **(A)** Conidia from strain *Δnox-1 nox-1-mCherry e* were induced to germinate and observed using confocal microscopy. NOX-1::mCherry is localized in round (white arrows) and tubular (yellow arrows) organelles. Images belong to a series of frames from a movie (not shown). **(B)** Conidia from strain *Δnox-1 nox-1-mCherry e* were induced to develop CATs and observed using confocal microscopy. Figures represent still images from Video S1. Left panel shows NOX-1-mCherry fluorescence signal of two conidia with CATs fused at the indicated point (white arrow). The right panel shows the same image merged to the corresponding bright field image.

When inoculated at high density, conidia from *N. crassa* and other fungi develop specialized structures, called conidial anastomosis tubes or CATs, for cell-cell fusion (Fischer and Glass, 2019). Although *Δnox-1* mutants differentiate CAT structures, these cannot undergo cell-cell fusion (Cano-Dominguez et al., 2008; Read et al., 2012). We used confocal microscopy to determine NOX-1::mCherry localization during germination, and to assess if this allele complemented *Δnox-1* cell fusion defects. Results in Figure 1A show that in germinating conidia the NOX-1::mCherry signal, largely absent from the hyphal tip, was localized in discrete round and tubular structures behind the hyphal tip, which moved very fast and showed very dynamic shape changes. CATs from strain *Δnox-1 nox-1::mCherry* were competent for cell-cell fusion (Figure 1B) and NOX-1::mCherry labeled structures were exchanged freely between the fusion partners (Video S1). This shows that NOX-1::mCherry expressed from *ccg*-1 promoter complements all *Δnox-1* mutant phenotypes, and its localization in mobile globular and filamentous structures during conidial germination.

### In Growing Hyphae NOX-1 Is Localized in Pleomorphic Structures Consistent With the Morphology of the Entire Vacuolar System

**Figure 2:**
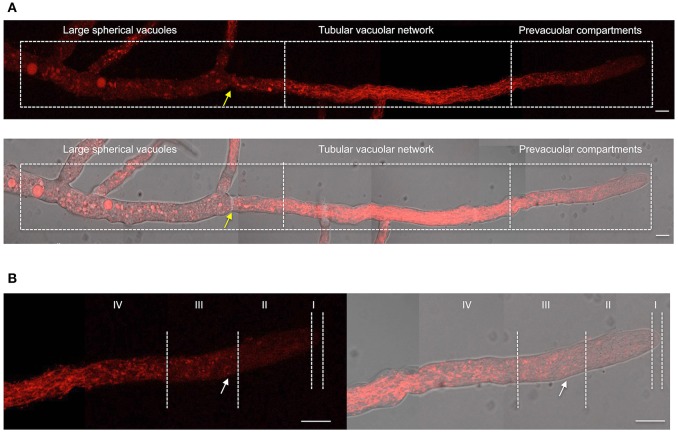
NOX-1 localization during hyphal growth. **(A)** A single hyphae from strain *Δnox-1 nox-1-mCherry e* composed using nine overlapping pictures obtained with fluorescent microscopy (upper panel) and the overlap with the bright field image (lower panel). **(B)** Enlargement of the prevacuolar compartment shown in **(A)** divided in subcompartments I to IV, using the hyphal tip as reference, according to Bowman et al. (2015). Yellow arrows indicate the position of the septum and the white arrow points to one of several u-shaped structures observed. White bars represent 10 μm.

Having shown NOX-1::mCherry fusion functionality, we next determined NOX-1 localization during hyphal growth using *in vivo* confocal microscopy. Figure 2 shows the reconstruction of a single hyphae in which three major characteristic regions can be distinguished; the actively growing tip, a central region and an older region that contains large spherical vacuoles. In contrast to the simpler pattern observed in germinated conidia (Figures 1A,B), NOX-1::mCherry signal displayed a highly differentiated pattern within these three regions. First, mCherry signal was very low closer to the hyphal tip and then gradually increased behind the tip, defining small round structures, some presenting u- or o-ring shapes (white arrow in Figure 2B). Second, the intensity and density of fluorescent signal increased toward the second hyphal segment up to the first septum, displaying a well-defined tubular network. Third, a more discrete pattern, including large round structures, was observed in the older region of the hyphae, where proteins that belong to the vacuolar system such as CAX, NCA-2, and some times VMA-1 have been localized (Bowman et al., 2009). Considering that the pattern of these three regions is notably similar to the one recently reported for the entire vacuolar system in *N. crassa* (Bowman et al., 2015), we also delimited them as the prevacuolar compartment, tubular vacuolar network and large spherical vacuolar regions, respectively (Figure 2A). In turn, the prevacuolar region was divided in regions I, II, III, and IV (Figure 2B), according to the population of vacuoles, distance from the hyphal tip and Bowman's nomenclature (Bowman et al., 2015). Figure 2B shows an enlarged image of the prevacuolar region shown in Figure 2A. As indicated before, NOX-1::mCherry signal was low in regions I and II. In zone III it became more visible, being located in small structures and some rings or u-shaped structures (Figure 2B and Video S2). Zone IV shows a transition between the morphology of zone III and the beginning of the dense network of interconnected filaments that extends backwards until the position of the first septum. These results are consistent with NOX-1 being localized in the entire vacuolar system.

### NOX-1 Shows Minor Co-localization With Endoplasmic Reticulum and Endosome Markers

**Figure 3:**
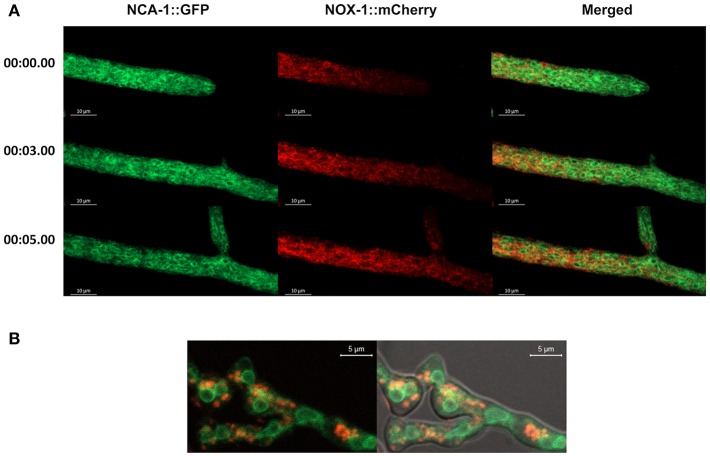
ER-marker NCA-1-GFP shows limited co-localization with NOX-1::mCherry. **(A)** Heterokaryotic hyphae from strains expressing NCA-1::GFP and NOX-1::mCherry were observed by confocal microscopy. Images correspond to time frames from a movie (not shown), as indicated in the left. **(B)** Conidia from NCA-1::GFP and NOX-1::mCherry strains were inoculated under CAT inducing conditions and heterokaryotic germlings observed by confocal microscopy.

In filamentous fungi *P. anserina* (Lacaze et al., 2015) and *Botrytis cinerea* (Siegmund et al., 2013, 2015), NOX-1 has been detected mainly in the ER and in vesicles proposed to derive from the ER (Schurmann et al., 2013; Siegmund et al., 2013, 2015; Lacaze et al., 2015). To explore this in *N. crassa*, we performed co-localization experiments following NOX-1::mCherry and NCA-1, a homolog of the SERCA-type Ca^2+^-ATPase, as an ER marker (Bowman et al., 2009), tagged with GFP. Heterokaryotic mycelia from NCA-1::GFP and NOX-1::mCherry strains were analyzed by confocal microscopy. Results in Figure 3A show that, as reported before, NCA-1 is observed as ring structures, previously shown to represent the nuclear envelope, distributed along the entire hyphae, including the growing tip. Although NOX-1 is also seen in some ring structures, these are fewer and smaller than NCA-1-labeled nuclear envelopes. Furthermore, double label experiments showed virtually no co-occurrence between NCA-1-GFP and NOX-1-mCherry labeled structures. If any, co-localization was limited to areas with high GFP and mCherry labeling density, while NOX-1 was mostly localized in filamentous structures and largely excluded from the hyphal tip. Likewise, NCA-1 and NOX-1::mCherry showed limited co-localization during CAT induction, where NCA-1 can be observed around putative nuclei and NOX-1 in round structures (Figure 3B). This suggested that *N. crassa* NOX-1 is not largely associated to the ER. To confirm this result, we generated strains in which the ER was labeled using a different marker. For this, we transformed wild-type strain 4200 with plasmid pAM01, encoding an ER-lumen targeted GFP (ER-GFP), containing ER-signal and ER-retention sequences from *P. anserina* BiP chaperone protein, a classical ER-marker (Pidoux and Armstrong, 1992; Plemper et al., 1997), fused to *gfp* and expressed from *A. nidulans gpdA* promotor (Meizoso-Huesca A. and Peraza-Reyes L., unpublished). Results in Figure S3, show that this protein labeled a reticular network along the entire hyphae, as well as putative nuclear envelope structures, consistent with ER labeling. The difference with NCA-1 ER signal, mostly enriched in putative nuclear envelope structures suggest that NCA-1 and ER-GFP might be enriched in different ER domains. As in the case of NCA-1 (Figure 3), NOX-1 presented very limited co-localization with ER-GFP labeled structures in both growing hyphae (Figure S3A) and under CAT inducing conditions (Figure S3B).

**Figure 4:**
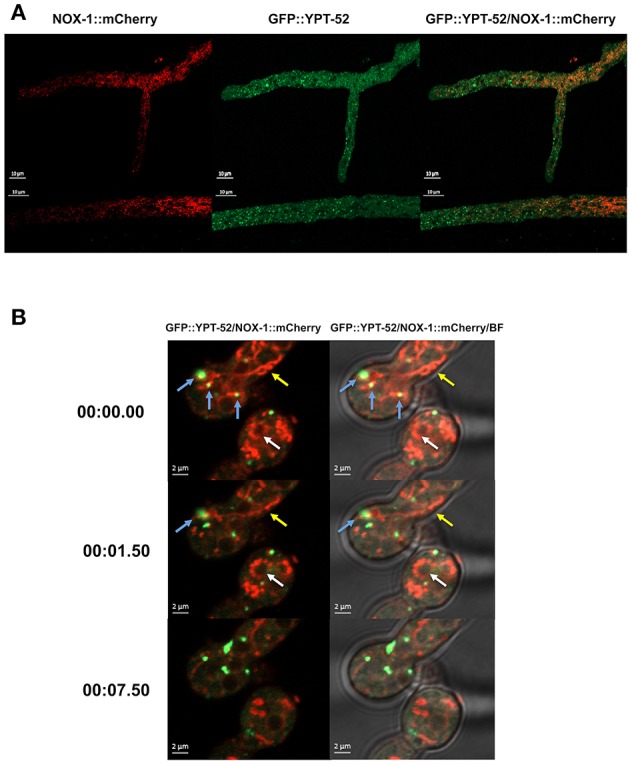
Early endosome marker GFP-YPT-52 shows minor co-localization with NOX-1::mCherry. **(A)** Heterokaryotic hyphae from strains expressing GFP-YPT-52 and NOX-1::mCherry were observed by confocal microscopy. **(B)** Conidia from strains expressing NOX-1::mCherry and GFP-YPT-52 were inoculated under CAT inducing conditions and heterokaryotic germlings were observed by confocal microscopy. Blue arrows indicate co-localization points between GFP-YPT-52 and NOX-1::mCherry, yellow arrows point to NOX-1::mCherry filamentous structures and white arrows point to NOX-1::mCherry structures arranged in a circular shape that change between continuous and dotted patterns (see corresponding Video S3).

To explore NOX-1 localization in early endosomes, defined as Rab5-labeled organelles, we produced heterokaryons between the NOX-1::mCherry strain and a strain containing the Rab5 GTPase early endosome protein YPT-52 labeled with GFP (Seidel et al., 2013). Figure 4A shows that as reported before, in growing hyphae YPT-52 shows a punctate pattern distributed along the entire hyphae, including the tip. Again, it was difficult to appreciate a co-localization of NOX-1 and YPT-52. Furthermore, YPT-52-stained puncta tended to accumulate toward hyphal apical segments in inverse correlation to NOX-1-labeled structures. However, some limited but clear co-localization was observed under CAT inducing conditions. Figure 4B and Video S3 show heterokaryotic conidia formed by cell-cell fusion, one of which shows three initial points of contact between endosomes and NOX-1-labeled organelles (blue arrows). Two of these contact points quickly disappear, while the upper one is maintained for a longer time and finally released. Notably, under these conditions NOX-1 labeled structures were highly pleomorphic, shifting between long filaments (yellow arrows) and dotted structures that were isolated or arranged in circles that assembled and disassembled (white arrows). Moreover, NOX-1 labeled structures seen first as a continuous circular filament a few seconds later become individual round structures, maintaining the circular arrangement (Video S3). In summary, these results show that NOX-1 is localized in structures that are consistent with the vacuolar system, and these are very dynamic and pleomorphic, changing their shape during hyphal aging, development, and cell fusion. Moreover, results indicate that NOX-1 shows limited co-localization with early endosomes and the ER.

### NOX-1 Co-localizes With Vacuolar ATPase VMA-1

**Figure 5:**
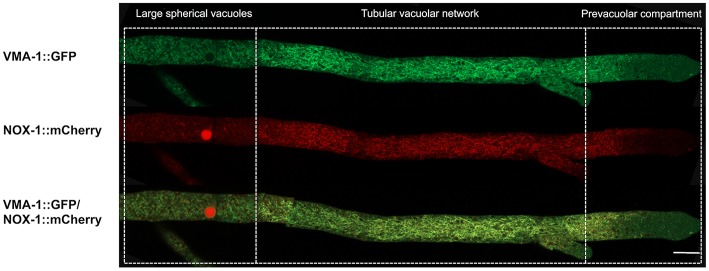
NOX-1::mCherry co-localizes with the vacuolar system marker VMA-1 ATPase. **(A)** Heterokaryotic hyphae from strains expressing NOX-1::mCherry and VMA-1::GFP were observed by confocal microscopy. Bar corresponds to 20 μm.

VMA-1 corresponds to subunit “A” of the vacuolar ATPase (V-ATPase). V-ATPases are membrane proteins that hydrolyze ATP to pump protons across the membrane and acidify the lumen of vacuoles and other cell compartments. In *N. crassa*, VMA-1 has been localized in the entire vacuolar system (Bowman et al., 2015), displaying a pattern very similar to the one we report here for NOX-1. To determine if these two proteins co-localize, a Δnox-1 NOX-1-mCherry*/*VMA-1-GFP heterokaryon was analyzed by confocal microscopy. As reported, we detected VMA-1 in ring and tubular structures that correspond to the vacuolar system. Notably, VMA-1 and NOX-1 co-localized in the same structures (Figure 5), except in the region of large spherical vacuoles in which NOX-1 was localized in the luminal part of the vacuoles and VMA-1 in the periphery.

**Figure 6:**
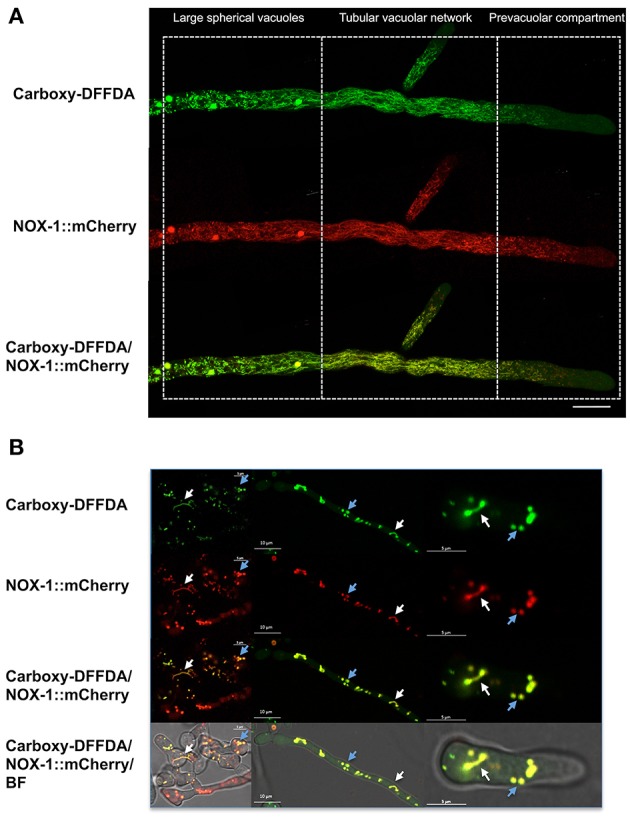
NOX-1::mCherry co-localizes with the vacuolar system marker carboxy-DFFDA. **(A)** Hyphae from strains expressing NOX-1::mCherry were stained with carboxy-DFFDA and observed by confocal microscopy. **(B)** Conidia from strain NOX-1::mCherry were induced to form CATs (left panels) or to germinate (middle and right panels), stained with carboxy-DFFDA, and observed by confocal microscopy.

To further confirm NOX-1 localization in the vacuolar system, we stained the *Δnox-1 NOX-1::mCherry* strain with carboxy-DFFDA, a molecule that accumulates in fungal acidic vacuolar compartments, producing a fluorescencent signal (Richards et al., 2012; Bowman et al., 2015; Rico-Ramirez et al., 2018). As shown in Figure 6A, NOX-1::mCherry localized in the same compartments stained by carboxy-DFFDA, including the luminal region of the large spherical vacuoles. The same co-localization was observed during conidia germination and CAT fusion, and as before, the shape of the vacuolar system was composed of larger and less filamentous structures (Figure 6B).

### NOX-1 Is Also Partially Localized at the Plasma Membrane

In plants, NOX AtrbohC has been detected at the plasma membrane of root hair cells, where it plays a critical role in polar growth (Takeda et al., 2008). We addressed the possibility that in our experiments NOX-1 membrane localization was being overshadowed by the strong NOX-1::mCherry organellar signal, derived from its constitutive expression. First, we used latrunculin A to inhibit the endocytic process, as a strategy to increase possible NOX-1 plasma membrane signal. As expected, latrunculin A delayed hyphal tip growth and under these conditions some NOX-1::mCherry signal was detected at the plasma membrane (not shown).

**Figure 7:**
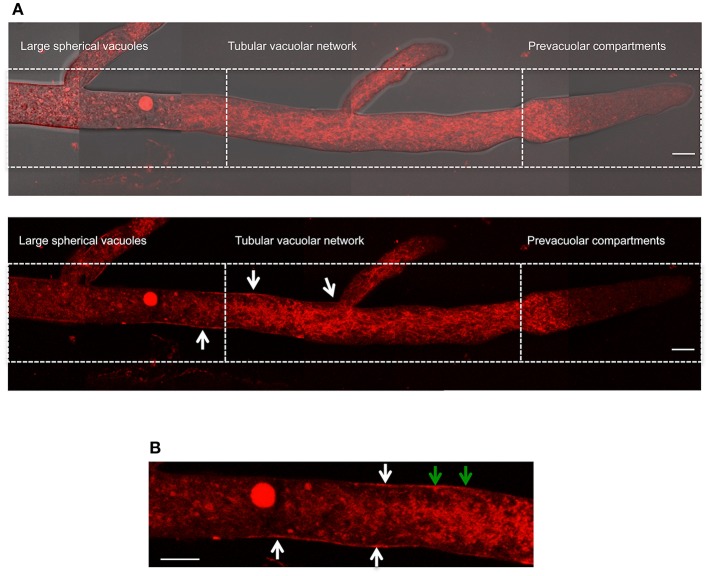
Localization of NOX-1::mCherry expressed from native *nox-1* promoter during hyphal growth. **(A)** Composition of a hypha using overlapping pictures obtained with fluorescent microscopy (lower panel) merged with the corresponding bright field image (upper panel), and divided in compartments as in Figure 2. The bars indicate 10 μm. **(B)** Enlargement of a region around the intersection between large spherical vacuoles and tubular vacuolar network compartments shown in **(A)** (lower panel). White arrows point to NOX-1::mCherry plasma membrane localization. Green arrows point to places that suggest endocytic activity. The bar indicates 10 μm.

To further confirm our NOX-1 localization results, we generated strains in which *nox-1::mCherry* was expressed from its native promoter. As shown in Figures S4A–E, expression of NOX-1::mCherry from *nox-1* promoter also resulted in full complementation of all *Δnox-1* defects. When strain *Pnox-1-nox-1::mCherry* was analyzed by confocal microscopy, it was evident that the fluorescence signal produced from the *nox-1* promoter was lower than the one produced from *ccg-1* promoter. Nevertheless, it could be appreciated that NOX-1::mCherry was largely absent from hyphal tips and formed a tubular network behind the tip, showing a vacuolar system pattern very similar to the one observed before (not shown). After increasing laser intensity, it was possible to confirm that NOX-1::mCherry signal also localized to the entire vacuolar system (Figure 7A), showing a pattern virtually identical to the one observed using the *ccg-1* promoter (Figure 2). Notably, under these conditions NOX-1 also was detected at the cell surface, consistent with plasma membrane localization. This was reproducibly observed in hyphal regions corresponding to the tubular vacuolar network and large spherical vacuole regions (Figures 7A,B). However, NOX-1 plasma membrane localization at the prevacuolar compartment region and hyphal tip was not as consistent, and only in some cases NOX-1 localized to the cell surface at the hyphal tip (Figure S3A, white arrows). After confirming that NOX-1::mCherry localization is alike whether expressed from *ccg-1* or *nox-1* promoters, we continued our NOX-1 localization studies using strains that expressed NOX-1::mCherry from *ccg-1* promoter, as their stronger signal allowed longer imaging acquisition and lower photo-bleaching.

### NOX-1 and Regulatory Subunit NOR-1 Co-localize Only at Very Discrete Compartments

While NOX-1 contains 6 putative transmembrane domains, its regulatory subunit NOR-1 lacks any clear signal peptide or transmembrane domain. However, since NOR-1 is required for NOX-1 function, we decide to compare the localization of both proteins. For this purpose, we generated a PCR construct coding for NOR-1 fused to GFP at the C-terminus and used it to transform strain FGSC-9718. Three homokaryotic strains were obtained in which the *nor-1::GFP* construct replaced the *nor-1* gene, as confirmed by Southern blot analysis (not shown). These strains showed a wild type phenotype, indicating that GFP tagging of NOR-1 did not affect its activity. However, when these strains were observed by confocal microscopy GFP signal was very low and difficult to detect (not shown). Therefore, we expressed *nor-1::GFP* from the *ccg-1* promoter as we did for NOX-1::mCherry. For this, we generated plasmid pNCNOR-1::GFP18 and used it to transform *Δnor-1* mutant strain Nc28nor-1. Ten transformants were obtained, from which GFP fluorescence positive strains were screened using epifluorescence microscopy. Two of these were confirmed by PCR (Figure S5) and used for NOR-1 localization experiments. This *nor-1::gfp* allele was able to restore all asexual, sexual and growth defects of *Δnor-1* mutants, indicating its full functionality (Figure S6).

**Figure 8:**
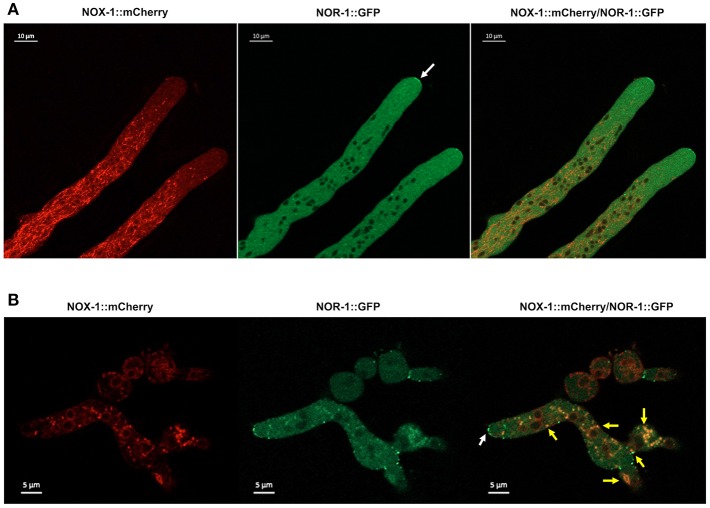
NOX-1::mCherry co-localizes with NOR-1::GFP during CAT inducing conditions. **(A)** Heterokaryotic hyphae from strains expressing NOX-1::mCherry and NOR-1::GFP were observed by confocal microscopy. A white arrow points to NOR-1::GFP enrichment at the hyphal tip. **(B)** Conidia from NOX-1::mCherry and NOR-1::GFP strains were incubated under CAT inducing conditions and observed by confocal microscopy. The white arrow indicates NOR-1::GFP localized at the hyphal tip in a dotted pattern and yellow arrows indicate single dot or dotted regions where NOX-1 and NOR-1 co-localize.

NOR-1::GFP fluorescence pattern was very different form the one observed for NOX-1. In growing hyphae, it was detected as a fine granular pattern along the entire hyphae and also in a few larger puncta. NOR-1 was clearly present in the hyphal tip, very likely at the plasma membrane and mainly confined to the apical dome (Figure 8A and Figure S7A). Notably, NOR-1 did not show any clear co-localization with NOX-1 in growing hyphae (Figure 8A) or germinating conidia (Figure S8B), where NOR-1::GFP was observed in punctate structures close to the plasma membrane (Figure S7C). In older hyphal regions (Figure S7B) and in intact conidia (Figure S8A), NOR-1::GFP displayed a punctuate pattern. In conidiophores (Figure S8A) both NOR-1 and NOX-1 showed the circular dotted structures described before for NOX-1 under CAT inducing conditions (Figure 4B). In some cases, NOR-1 and NOX-1 dotted structures seemed to be intercalated and in few cases the two signals co-localized, suggesting that NOR-1 and NOX-1 localize to specific domains of these structures, where they can eventually converge. Because both NOX-1 and NOR-1 are essential for CAT fusion, we incubated conidia from NOX-1::mCherry and NOR-1::GFP strains at high density and made observations after 4 h, where profuse CAT fusions take place. Under these conditions, we observed both clear segregation and co-localization of NOR-1 and NOX-1 signals. Figure 8B shows three heterokaryotic conidia (i.e., after cell fusion) at different germination stages. The two top conidia show a NOX-1 pattern, consisting of strands and puncta located within cells and at the cell surface. In addition, NOX-1 also decorated the periphery of what appears to be large vacuolar structures. In these conidia NOR-1::GFP displays a different pattern, with isolated puncta located at the cell surface and a septum. In the conidium at the bottom of the figure, which is in a more advanced stage of germination and has fused with a contiguous conidium, NOX-1 localization pattern is similar to the one described above. However, NOR1-GFP more conspicuously stained the hyphal tip at this stage (white arrow), and clear co-localization of NOR-1 and NOX-1 was observed in puncta located at the cell surface, the cytoplasm and the cell-cell fusion interface, as well as some associated with putative large vacuoles (yellow arrows). Together, these results suggest a dynamic localization of NOR-1 and NOX-1 at specific domains of the plasma membrane and the cytoplasm–notably including the vacuolar system–, where they interact at specific compartments, which are enriched during the cell-fusion interactions that occur under CAT inducing conditions.

### NOX-1 Single Amino Acid Substitutions in NADPH-Binding Sites Result in a Complete Lack of Function

We took advantage of the functionality of NOX::mCherry to evaluate the effects of specific amino acid substitutions in NOX-1 function. The analysis of mutations in human CYBB gene, encoding gp91phox catalytic subunit, or NOX2, showed that the substitution of proline 415 by histidine resulted in a lack of oxidase enzyme activity but preservation of the inactive protein. The same was observed with mutations replacing arginine 537 by cysteine (Rae et al., 1998). These amino acids, which are part of the NADPH-binding sites, are highly conserved in the NOX from filamentous fungi (Figure S9), and correspond to NOX-1 proline 382 and cysteine 524. We used plasmid pNCNOX-1::mCherry4 to introduce point mutations by PCR in the corresponding codons, to generate NOX-1-mCherry proteins with P382H and C524R substitutions. The resulting plasmids were confirmed by DNA sequencing and used to transform *Δnox-1 his-3* strain. Two transformants confirmed by PCR were named *NOX-1P382H-mCherry* *Δnox-1* and *NOX-1C524R-mCherry* *Δnox-1* and used for further experiments. The analysis of the corresponding strains by confocal microscopy showed that the signal intensity and localization of the mutant proteins was very similar to wild type NOX-1 (Figure S10). Nevertheless, *nox-1*^*P*382*H*^*-mCherry* and *nox-1*^*C*524*R*^*-mCherry* alleles were unable to complement any of the defects observed in *Δnox-1* mutants (Figures S11A–F). These results indicate that all NOX functions depend on its oxidase activity.

## Discussion

### NOX-1 Is Localized in the Vacuolar System

The localization of a protein is a determinant of its functions. Previous reports on NOX subcellular localization in different filamentous fungi concluded that these enzymes are mainly associated to the ER. In *P. anserina*, PaNox1-GFP tagging resulted in a reticulate pattern, in some cases perinuclear, showing high co-localization with an ER marker in young hyphae, while in older hyphae a cortical punctuated pattern was more frequent. The authors concluded that PaNox1 is mainly localized in the ER, as well as in cortical vesicles originated from the ER and presumably involved in the vacuolar network (Lacaze et al., 2015). However, the ER marker consisted of mCherry tagged with the predicted secretion signal from NOX subunit PaNoxD and the KDEL ER-retention signal, and no other independent ER marker was used.

In *B. cinerea*, BcNoxA and BcNoxB GFP fusions expressed constitutively were localized in similar structures in germinated conidia. Both GFP-NoxA and NoxB-GFP showed partial co-localization with an ER-tracker signal, in some cases in structures surrounding nuclei (Siegmund et al., 2013). In *Claviceps purpurea*, mCherry fusions of CpNox1 and CpNox2, not tested for functionality, were expressed constitutively and localized using epifluorescence microscopy. Using ER-Tracker Blue-White DPX, these authors concluded that CpNox1 and CpNox2 appeared to be located mainly in the ER while CpNox2 also showed a tendency to accumulate in unspecified vacuoles (Schurmann et al., 2013). In support of ER localization, Siegmund et al. (2015) showed that a *B. cinerea* GFP::BcNoxA fusion designed to be artificially retained in the ER, by addition of the HDEL motif, restored the pathogenicity defects of a *ΔbcnoxA* mutant. However, the fact that this fusion failed to complement *ΔbcnoxA* CAT fusion and sclerotia formation defects, led the authors to propose that BcNoxA has functions both inside and outside the ER (Siegmund et al., 2015). However, the possibility that GFP::BcNoxA::HDEL could leak from the ER cannot be excluded, as it is known that some KDEL bearing proteins are localized outside the ER (Soares Moretti and Martins Laurindo, 2017). In search of possible NOX functions at the ER, Marschall and Tudzynski (2017) detected physical interaction between BcNoxA and putative ER disulfide isomerase protein BcPdi1, and showed that *ΔbcnoxA* and *Δbcpdi1* mutants display similar phenotypes (Marschall and Tudzynski, 2017). However, there is increasing evidence indicating that disulfide isomerases can be located and have functions outside the ER (Soares Moretti and Martins Laurindo, 2017).

Our results using confocal microscopy show that functional versions of *N. crassa* NOX-1, expressed either from a constitutive promoter or from its own promoter, display limited co-localization with the ER and early endosomes and are mainly associated to the entire vacuolar system, as defined by the organellar system labeled by the vacuolar ATPase VMA-1 and the fluorescent molecule carboxy-DFFDA. The fact that in older hyphal regions the NOX-1::mCherry signal is located in the lumen of large vesicles suggests that NOX-1 degradation might occur in these cell compartments, as it has been observed for other vacuolar membrane proteins such as NCA-2, NCA-3, and CAX but not for VMA-1, which does not contain transmembrane domains (Bowman et al., 2009). In addition, NOX-1 proteolysis could result in cleavage of the mCherry tag. Although the reasons for these differences in fungal NOX localization (mainly ER *vs*. mainly vacuolar) are not known, they might be related to (i) the biological dissimilarity between *N. crassa* and the other fungi studied; (ii) the fact that different cell types or regions were studied in different fungi or (iii) technical and methodological reasons.

As shown before (Bowman et al., 2015; Rico-Ramirez et al., 2018) and in this work, *N. crassa* vacuolar system is a highly dynamic and pleomorphic structure, showing dramatic changes in shape during growth, aging, and development. In growing hyphae, the tubular vacuolar region has also been named as network of elongated cisternae or NEC. Studying the co-expression of CSE-7, a chaperone required for ER exit of some proteins, and different ER markers, Rico-Ramirez et al. (2018) concluded that what has been defined as ER and NEC show some overlap. How extensive this overlap is, structurally as well as functionally, is yet to be defined. However, it should be noted that while the subcellular localization of proteins depends mainly on their targeting signals, the accumulation of DFFDA and its fluorescence depends on specific transporters and an acidic pH, both associated with vacuolar compartments and not with the ER. Indeed, DFFDA never stains the nuclear envelope or the hyphal tip regions, while ER markers are readily detected in these regions. As NOX-1, VMA-1, and DFFDA largely stain the same compartments, we conclude that such compartments correspond to the vacuolar network. Interestingly, NOX2 and V-ATPase not only are both located in mammalian phagosomes, but V-ATPase activity is required for NOX2 oxidase activity (Rybicka et al., 2012).

Fungal vacuoles have functions similar to animal lysosomes and plant vacuoles (Richards et al., 2012). They play essential roles in protein and organelle degradation, xenobiotic detoxification, cellular pH, and osmotic homeostasis, and the storage of metabolites and critical ions like iron and calcium. We propose that some of these processes are ROS-regulated by NOX enzymes capable to produce low levels of ROS in the vacuolar lumen (see below), as exemplified by the redox regulation of cysteine cathepsin proteases in phagosomes (Rybicka et al., 2012).

### NOX-1 Is Also Localized at the Plasma Membrane

Although in growing hyphae most of the NOX-1-mCherry fluorescence signal was associated to the vacuolar system, we also detected part of NOX-1 at the plasma membrane, particularly in areas that correspond to the tubular vacuolar network and large spherical vacuoles regions. Polarized growth in plants has been associated with plasma membrane enrichment of specific NOX enzymes at the tip of root hair cells (Takeda et al., 2008). As *Δnox-1* mutants are affected in radial growth and growth of aerial mycelium, this suggests that NOX-1 plasma membrane localization might be linked to polar growth in this fungus. However, NOX-1 localization at the plasma membrane of the hyphal tip was observed only in some instances, including when latrunculin was used to partially inhibit endocytosis, and even in these cases the enzyme was not particularly enriched at the tip. This might be related to the fact that this region moves out of focus during growth, or it could indicate that NOX-1 membrane localization at the hyphal tip is transient and highly dynamic. In contrast, the NOX-1 regulatory subunit NOR-1 was indeed enriched at the hyphal tip (see below). In *M. oryzae*, using a NOX1-GFP fusion expressed from its own promoter and epifluorescence microscopy, Egan et al. (2007) detected faint GFP fluorescence at the periphery of appressoria, suggesting that NOX1 was localized at the plasma membrane. Notably, this fusion was also partially localized in the appressorium central vacuole, but its localization was not studied in other conditions. Although in *B. cinerea*, NoxA and NoxB were associated to the ER, faint fluorescence was also detected at the plasma membrane (Siegmund et al., 2013). It is not uncommon that only a portion of the total amount of cellular NOX is localized at the plasma membrane. In resting dendritic cells, only about 10% of total NOX2 is present on the plasma membrane and this increases to about 22% after zymosan stimulation, while a significant amount of this enzyme is detected in late endosomes and lysosomes (Dingjan et al., 2017).

### NOX-1 and NOR-1 Co-localize Only in Some Specific Compartments

As *Δnor-1* mutant phenotypes recapitulate the phenotypes of both *Δnox-1* and *Δnox-2* mutants, it has been considered that NOR-1 is necessary for the activity of both enzymes. Therefore, it was unexpected to find that NOX-1 and NOR-1 co-localize in only some cell compartments. Our NOR-1 localization results are largely consistent with the localization of NOR-1 orthologs in other fungi. In the plant symbiont *Epichloë festucae* grown in axenic culture, a NoxR-GFP fusion, not tested for functionality and expressed from a constitutive promoter, was detected in septa and hyphal tips showing both punctuated and more homogeneous patterns behind the tip. The hyphal tip localization depended on the presence of NoxR PB1 domain (Takemoto et al., 2011). In *B. cinerea*, a constitutively expressed functional GFP-BcNoxR fusion was detected in granules irregularly distributed throughout the vegetative hyphae. In some cases these granules were moving toward the hyphal tip, and no co-localization with BcNoxA or BcNoxB was observed (Siegmund et al., 2013). In *C. purpurea* a CpNoxR::mCherry fusion, not tested for functionality and expressed constitutively was detected equally distributed in the cytoplasm as well as within mobile particles (Schurmann et al., 2013). In *P. anserina*, PaNoxR was detected in a diffuse cytoplasmic pattern in young hyphal regions and in a punctuated pattern in older regions. In some cases PaNoxR was detected at the tip of growing hyphae, as well as the tip of hyphae undergoing anastomosis, and PaNox1 and PaNoxR co-localization was only observed in vesicles and never at ER-type structures or the hyphal tip (Lacaze et al., 2015).

We found that NOX-1 and NOR-1 co-localization is mainly observed in vesicular structures formed during CAT formation and cell-cell fusion conditions. It is possible that all the defects observed in *Δnor-1* and *Δnox-1* mutants could be explained by their inability to undergo cell fusion, both during hyphal growth and CAT fusion. Indeed, many mutants screened as defective for cell fusion were also affected in radial growth, aerial mycelium growth and were female sterile during sexual differentiation (Fu et al., 2011). CAT development is a complex process and more than 70 genes involved in cell recognition, chemotropic interactions and cell fusion have been identified in filamentous fungi. Among these NOX-1 and NOR-1 are essential for cell fusion in different fungi [see (Fischer and Glass, 2019) for a comprehensive review]. Interestingly, we have found that *Δnox-1* conidia cannot fuse with wild type conidia, indicating that a functional NOX-1 is needed in the fusing cells and highlighting the importance of NOX-1 during this process.

### NOX-1 Oxidase Activity Is Essential to Regulate Growth and Development in *N. crassa*

We have demonstrated that the substitution of a single amino acid in two different NADPH-binding sites is enough to render NOX-1 inactive. This indicates that the oxidase activity of NOX-1 is essential for its functions, as opposed to NOX-1 possible roles as a scaffolding protein, capable to interact with several proteins. NOX transport of the electrons taken form NADPH across a membrane not only produces superoxide and derived ROS, but can also affect the pH on either side of the membrane. This, in turn, affects electrochemical-driven ion fluxes and cell turgor (Segal, 2016). Because these two functions cannot be separated, the critical question on which NOX functions are attributed to the production of ROS and which to the associated changes in pH remains unanswered and therefore, it cannot be ignored that NOX enzyme function has these two effects.

The fact that electron transport activity can be induced *in vitro* by adding NADPH to purified and relipidated NOX2 (Koshkin and Pick, 1993) indicates that there is a low basal activity that seems independent of its regulatory subunits. Therefore, it is possible that the NOX-1 we observe in the vacuolar network could have some NOR-1 independent activity in this cell compartment, which might be important to regulate the acidic pH and contents of the vacuolar system, as well as cell turgor.

In addition, some NOX-1 targeted to the plasma membrane might be endocyted and fused with NOR-1 containing vesicles to generate a fully active enzyme and produce high ROS levels inside these vesicles. Some of these vesicles could travel back to the plasma membrane and release ROS to the extracellular space, where they could participate in cell-wall remodeling, defense and cell-cell communication (Aguirre and Lambeth, 2010). Some of these vesicles containing fully active NOX-1 could also be directed to specific cell compartments, where they could fuse and release ROS, as a way of generating different oxidation conditions in time and space.

In dendritic cells, where a sustained production of ROS is needed in the phagosome, it has been recently shown that lysosomes contain a large portion of inactive NOX2, which is recruited to the phagosome to replenish inactive oxidized NOX2 (Dingjan et al., 2017). Therefore, it is possible that part of the NOX-1 present in the fungal vacuolar system could also be a NOX-1 reservoir to be directed to NOR-1-containing or other cell compartments.

## Data Availability

The raw data supporting the conclusions of this manuscript will be made available by the authors, without undue reservation, to any qualified researcher.

## Author Contributions

JA designed experiments, wrote the manuscript, and obtained funding. NC-D performed and designed experiments, and contributed to manuscript writing. BB and LP-R contributed materials, and to discussion and manuscript writing.

### Conflict of Interest Statement

The authors declare that the research was conducted in the absence of any commercial or financial relationships that could be construed as a potential conflict of interest.

## Supplementary Material

Video S1 Conidia expressing NOX-1::mCherry undergoing cell-cell fusion.

Video S2 Growing hyphae expressing NOX-1::mCherry.

Video S3 Heterokaryotic conidia expressing endosome marker GFP-YPT-52 and NOX-1::mCherry under CAT inducing conditions.
