# Supplementary material for: Neurospora crassa NADPH Oxidase NOX-1 Is Localized in the Vacuolar System and the Plasma Membrane
Source: Front Microbiol. 2019 Aug 14;10:1825. doi: 10.3389/fmicb.2019.01825 (PMC6702951; doi:10.3389/fmicb.2019.01825)
Supplement: Supplementary file 4 [file Data_Sheet_2.pdf]

A

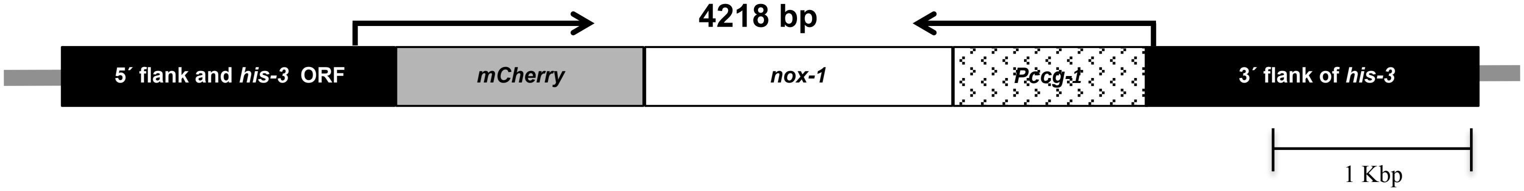

B

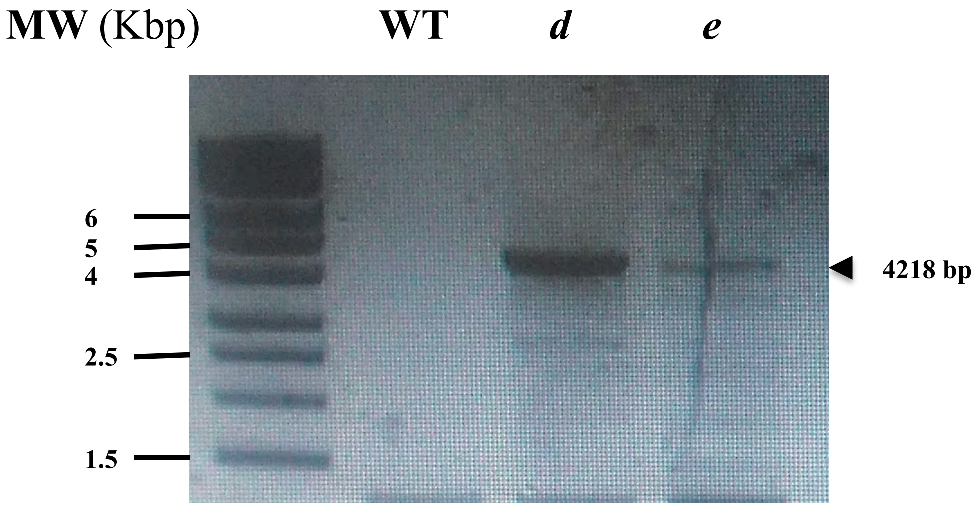

Figure S1. PCR confirmation of strains containing plasmid pNCNOX-1::mCherry4. (A) Plasmid pNCNOX-1::mCherry4 used to transform strain *Δnox-1 his-3* integrated at the *his-3* locus. **(B)** DNA from wild type (**WT**) strain and transformants *Pccg-1-nox-1-mCherry* Δ*nox-1d* (***d***) and *Pccg-1-nox-1-mCherry* Δ*nox-1e* (***e***) was used as template to amplify a 4218 bp fragment, using primers Hyperccg-1 secuenciarR and His-3F (Table S3). **MW** indicates molecular weight markers.

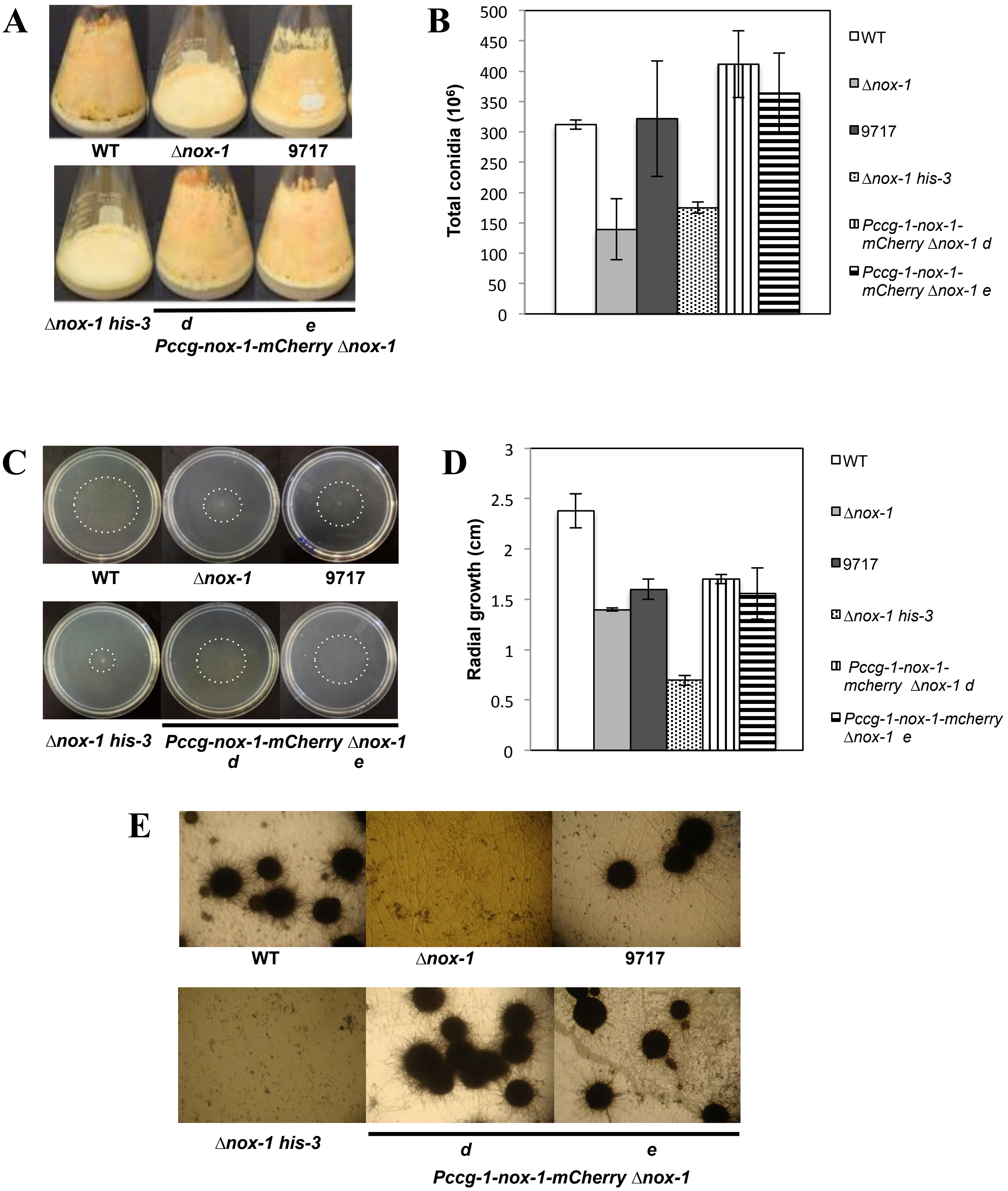

Figure S2. NOX-1::mCherry expressed from *ccg*-1 promoter complements *Δnox-1* mutant defects. (A) Indicated strains were inoculated ($1X10^4$ conidia) and incubated for 3 days in darkness and 2 days in light at 30 °C. **(B)** 1×10^4 conidia were inoculated in test tubes containing 3 mL of media and incubated as in **A,** and conidia were harvested and counted. Average of three independent experiments each with triplicates for each strain. **(C)** $1X10^3$ conidia were inoculated on solid media, incubated at 30 °C in the dark for 24 h, and radial growth of the colonies was measured **(D)**. Average of three independent experiments each with triplicates for each strain. **(E)** Indicated strains were induced to develop protoperithecia for 6 days, fertilized with WT conidia of the opposite mating type and incubated for 10 days at 25 °C.

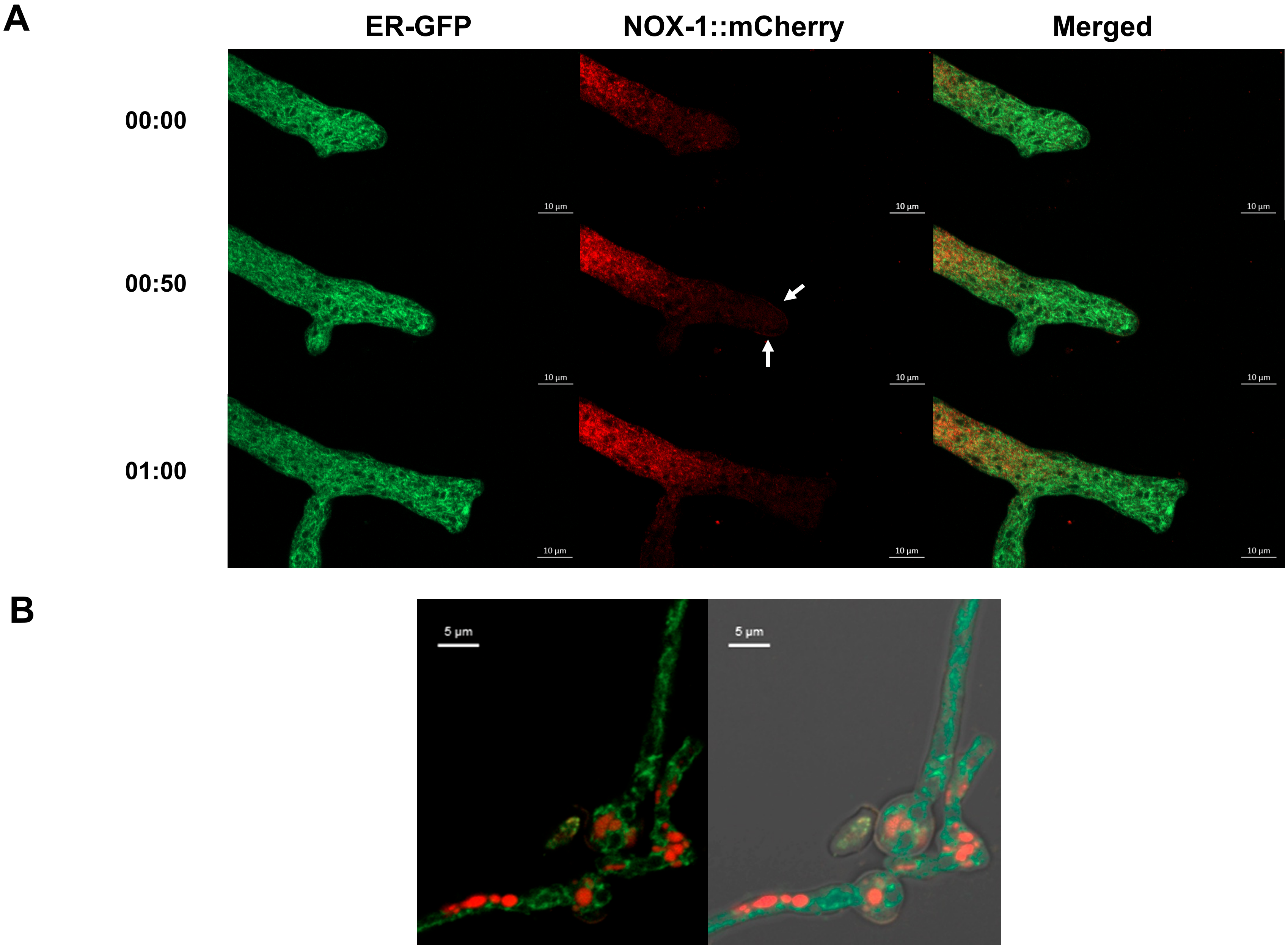

Figure S3. ER-marker GFP shows limited co-localization with NOX-1::mCherry. (A) Heterokaryotic hyphae from strains expressing ER-GFP and NOX-1::mCherry were observed by confocal microscopy. Images correspond to time frames from a movie (not shown), as indicated in the left. White arrows point to NOX-1::mCherry faint signal at the hyphal tip. **(B)** Conidia from strains expressing NOX-1::mCherry and ER-GFP were inoculated under CAT inducing conditions and heterokaryotic germlings were observed by confocal microscopy.

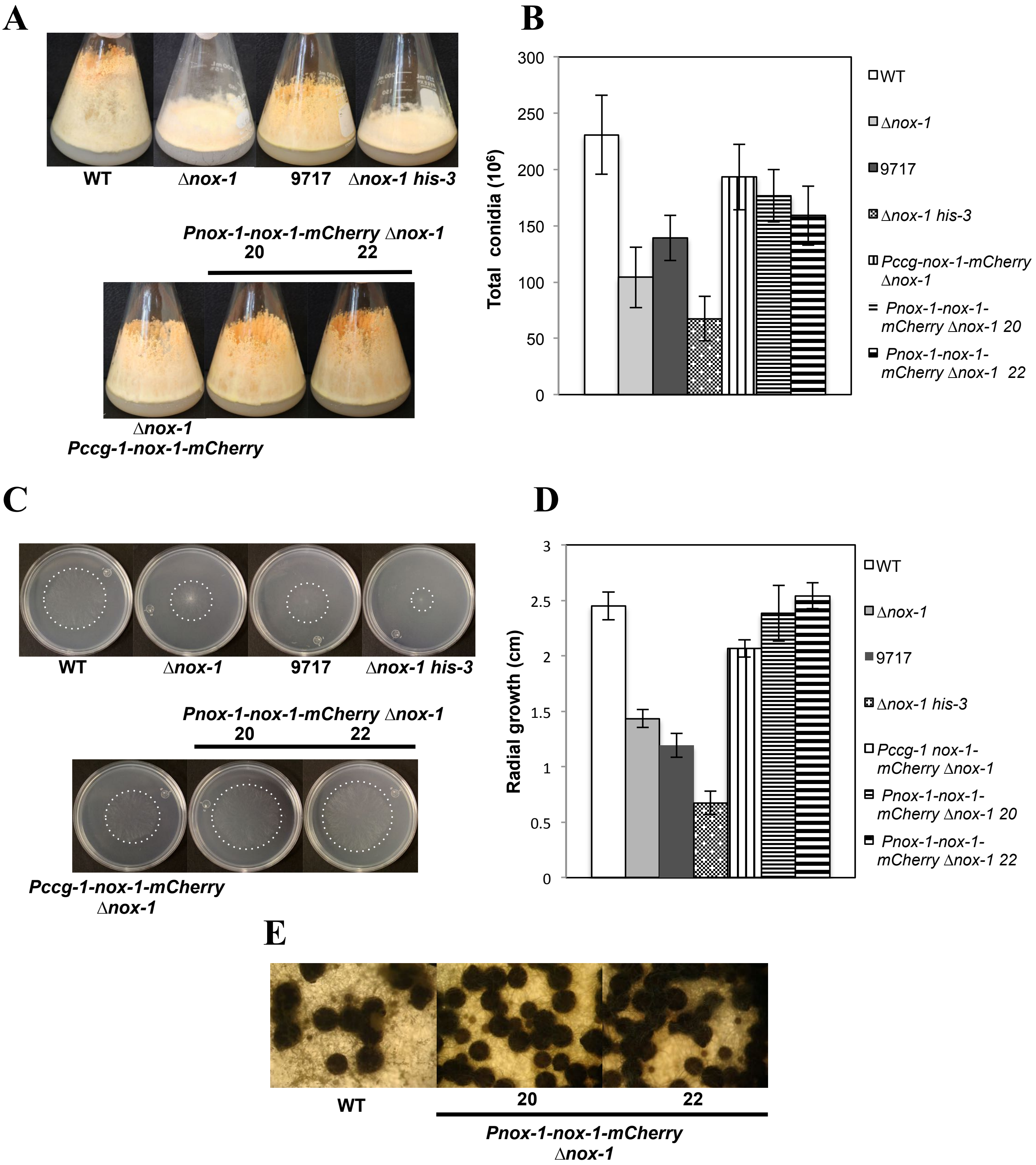

Figure S4. ***nox-1::mCherry*** **expression from its native promoter complements the defects of a Δ*nox-1* mutant.** (**A**) Indicated strains were inoculated (1X10^4 conidia) and incubated for 3 days in darkness and 2 days in light at 30 °C. (**B**) 1X10^4 conidia were inoculated in test tubes containing 3 mL of media and incubated as in **A** and conidia were harvested and counted. Average of three independent experiments each with triplicates for each strain. (**C**) 1X10^3 conidia were inoculated on solid media, incubated at 30 °C in the dark for 24 h, and radial growth of the colonies was measured (**D**). Average of three independent experiments each with triplicates for each strain. (**E**) Indicated strains were induced to develop protoperithecia for 6 days, fertilized with WT conidia of the opposite mating type and incubated for 10 days at 30 °C.

A

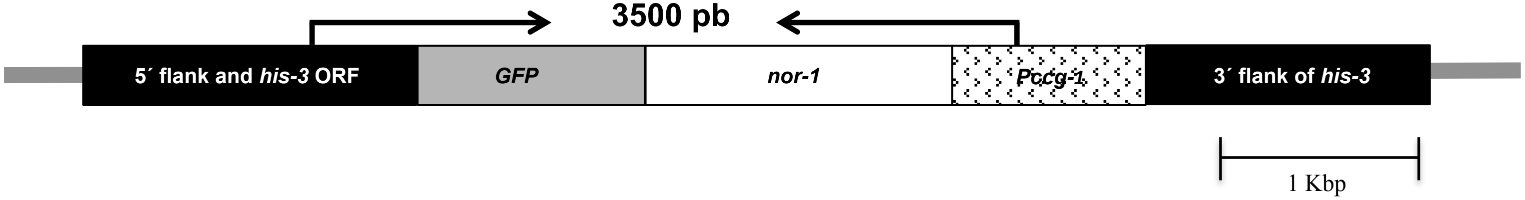

B

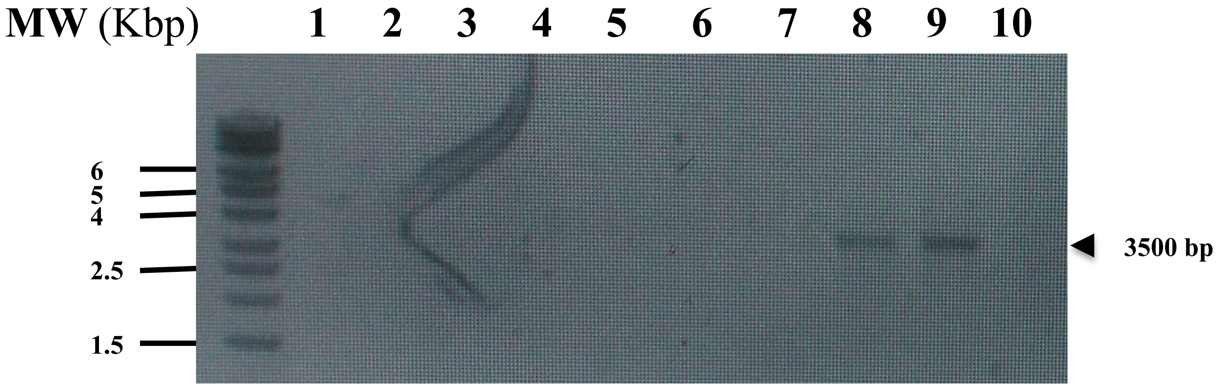

Figure S5. PCR confirmation of strains containig plasmid pNCNOR-1::GFP 18. (**A**) Plasmid pNCNOR-1::GFP18 used to transform Δ*nor-1*strain NcNor-28 integrated at the *his-3* locus. (**B**) DNA from transformants *Pccg-1-nor-1-gfp* Δ*nor-1* was used as template to amplify a 3500 bp fragment (8 and 9), using primers Hyperccg-1 secuenciarF and His-3R (Table S3). **MW** indicates molecular weight markers.

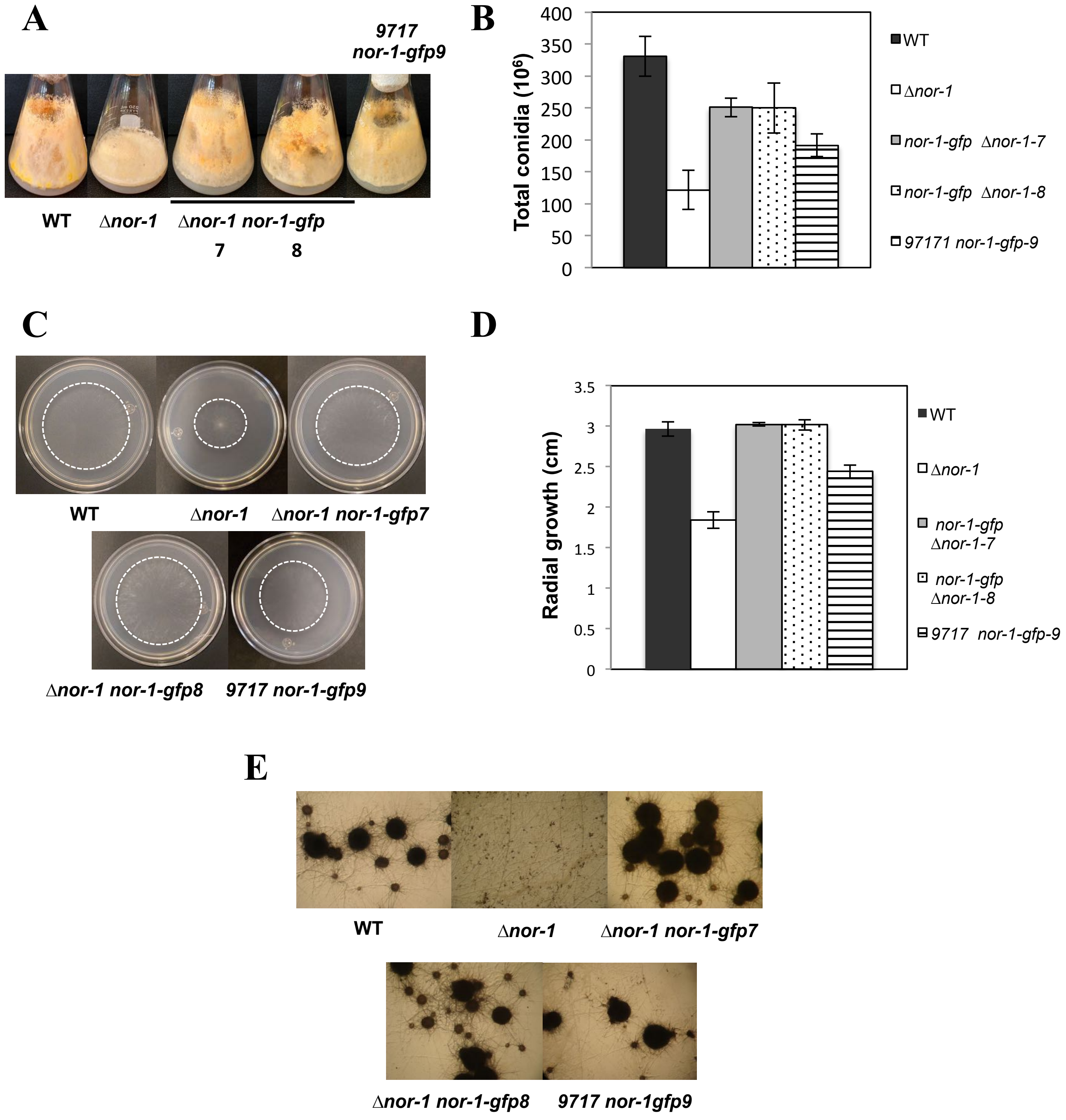

Figure S6. NOR-1::GFP expressed from *ccg*-1 promoter complements *Δnor-1* mutant defects. (**A**) Indicated strains were inoculated ($1X10^4$ conidia) and incubated for 3 days in darkness and 2 days in light at 30 °C. (**B**) $1X10^4$ conidia were inoculated in test tubes containing 3 mL of media and incubated as in **A** and conidia were harvested and counted. Average of three independent experiments each with triplicates for each strain. (**C**) $1X10^3$ conidia were inoculated on solid media, incubated at 30 °C in the dark for 24 h, and radial growth of the colonies was measured (**D**). Average of three independent experiments each with triplicates for each strain. (**E**) Indicated strains were induced to develop protoperithecia for 6 days, fertilized with WT conidia of the opposite mating type and incubated for 10 days at 25 °C.

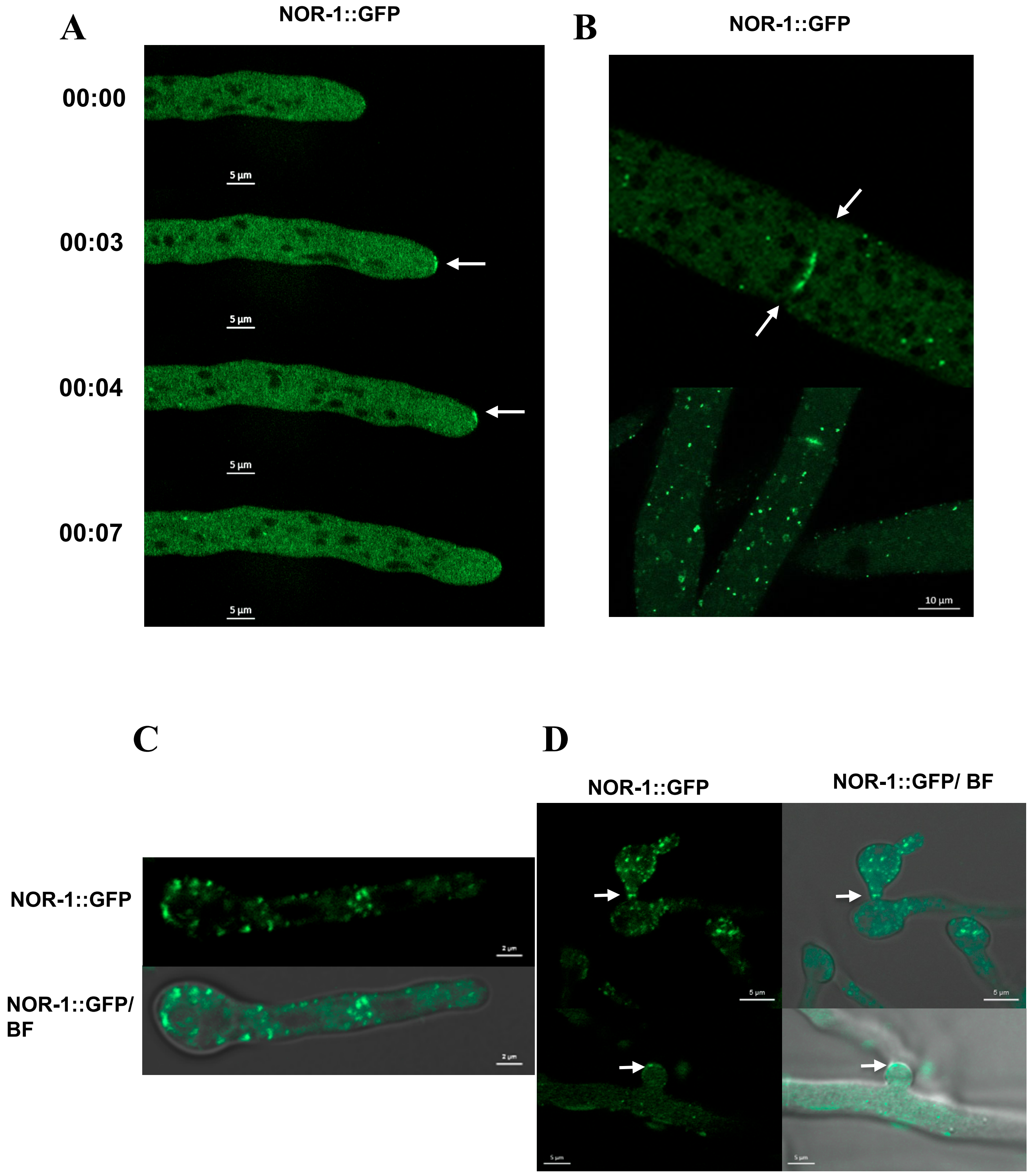

Figure S7. NOR-1::GFP localization during growth and CAT inducing conditions. (**A**) In growing hyphae (time indicated in the left) NOR-1::GFP shows a fine granular pattern and accumulates at the hyphal tip (arrows). (**B**) In older hypahl regions NOR-1::GFP forms larger granules, some times at the septum (arrow). (**C**) NOR-1::GFP larger puncta are also observed during conidia germination and at points of conidial (**D**, arrows in top panels) and hyphal fusion (**D**, arrows in lower pannels)

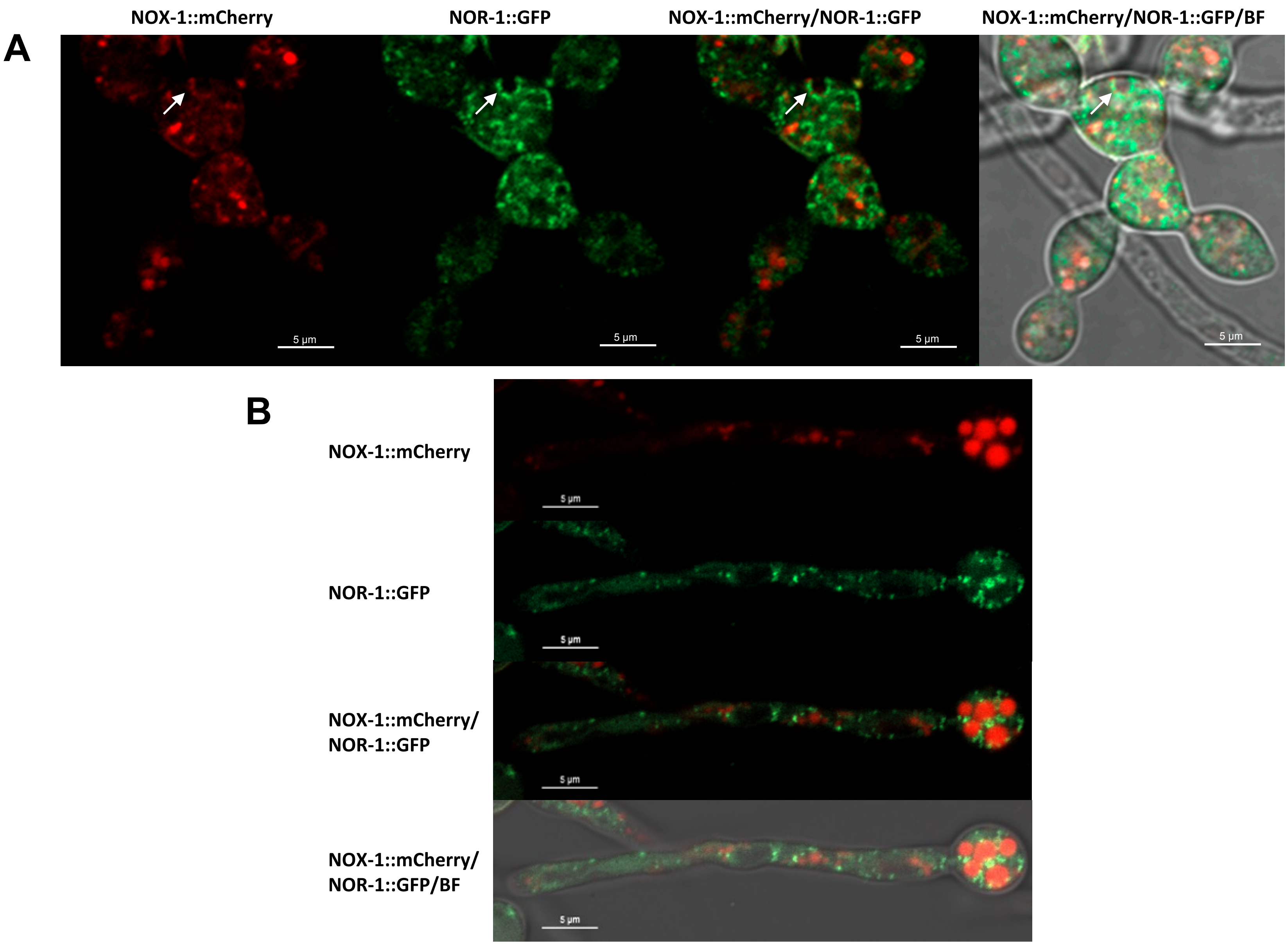

Figure S8. NOR-1::GFP and NOX-1::mCherry localization during conidiation and conidia germination. (**A**) Intact conidiophores from heterokaryons expressing NOR-1::GFP and NOX-1::mCherry were observed using confocal microscopy. (**B**) Conidia from heterokaryons expressing NOR-1::GFP and NOX-1::mCherry were germinated for 5 h and observed using confocal microscopy.

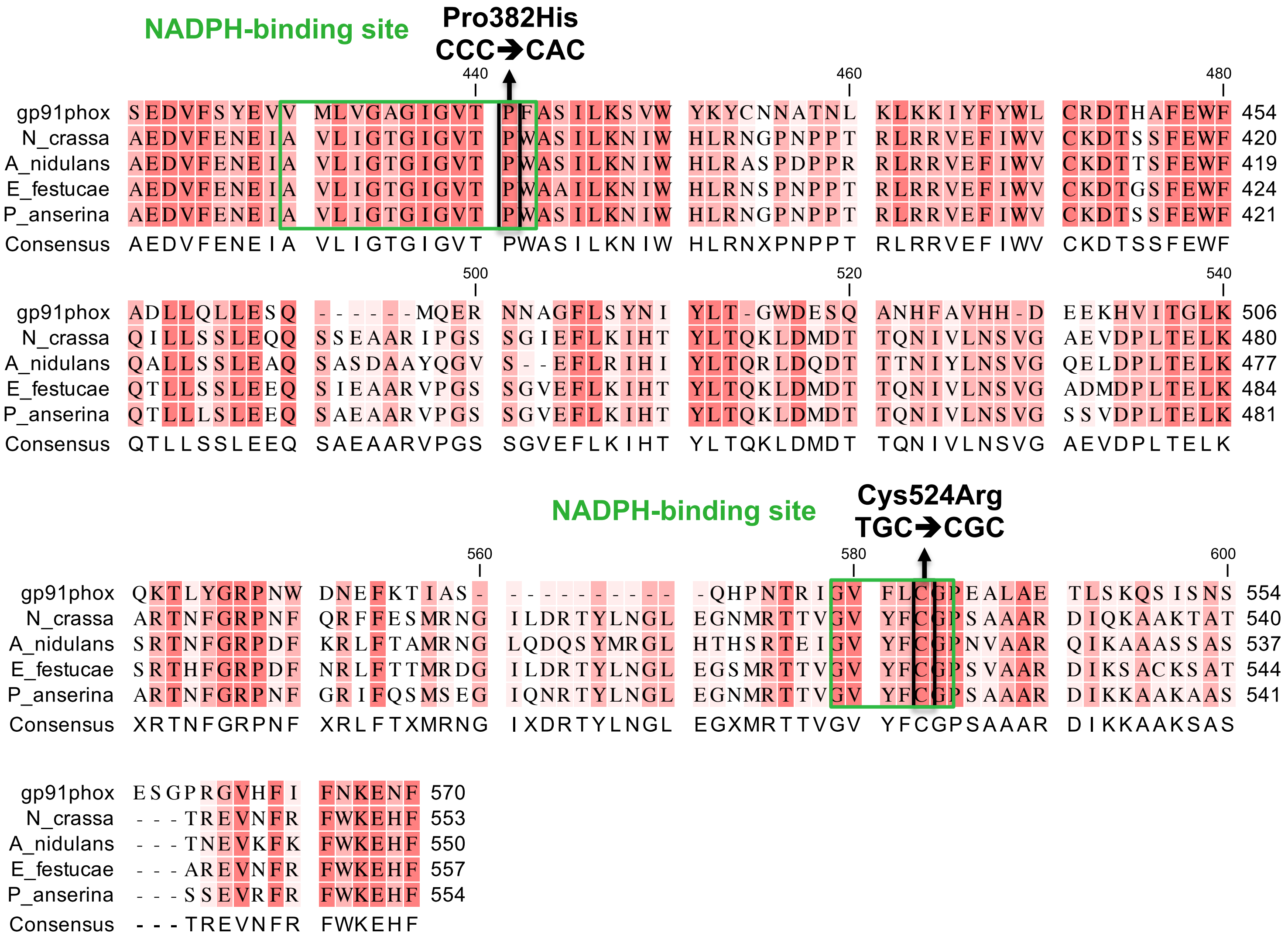

Figure S9. ***N. crassa*** **NOX-1 proline 382 and cysteine 524 are highly conserved amino acids in NADPH-binding domains of NOX proteins.** *H. sapiens* gp91phox (AKI71276) was aligned to *N. crassa* NOX-1 (EAA34868), *A. nidulans* NoxA (AAN75017), *E. festucae* NoxA (BAE72680) and *P. anserina* PaNox1 (AF364817) sequences. *N. crassa* NOX-1 proline 382 and cysteine 524, corresponding to human gp91phox (Nox2) proline 415 and cysteine 537, were replaced by histidine and arginine in mutants *NOX-1P382H::mCherry Δnox-1* and *NOX-1C524R::mCherry Δnox-1*, respectively.

A **B**

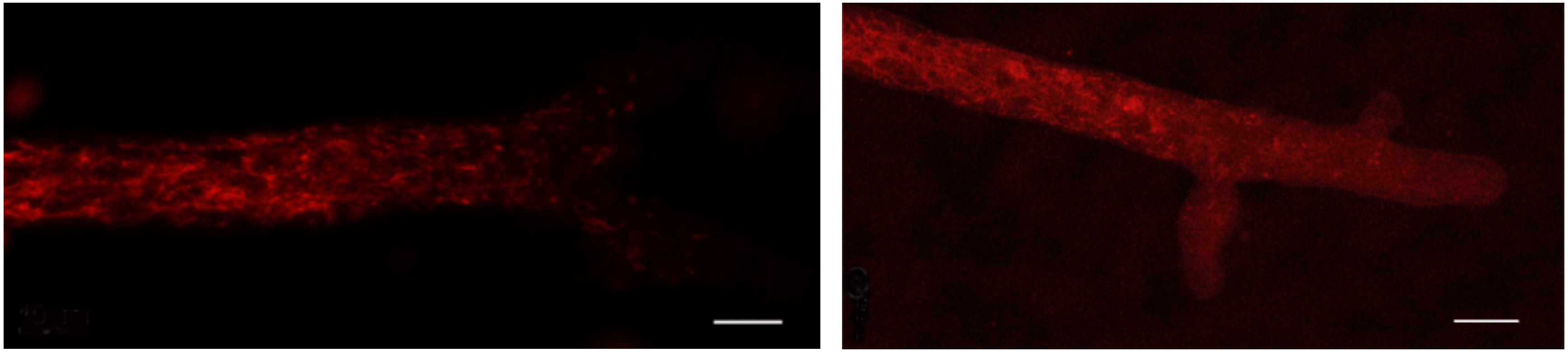

NOX-1P382H::mCherry Δnox-1 ***NOX-1C524R::mCherry Δnox-1***

Figure S10. NOX-1 single amino acid substitutions in NADPH-binding sites result in a complete lack of function without affecting subcellular localization. Hyphae from strains NOX-1P382H::mCherry Δnox-1 (B) were observed by confocal microscopy

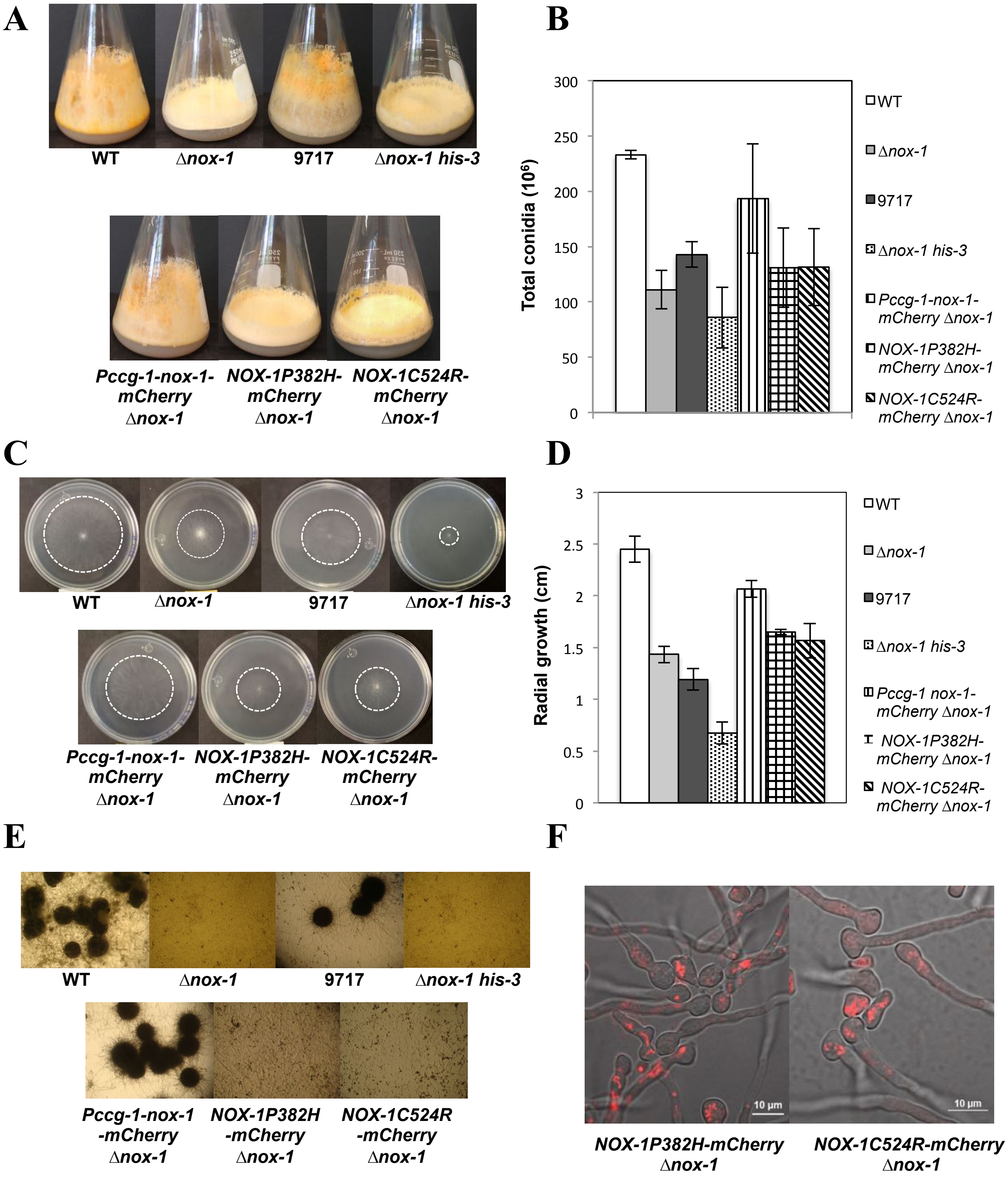

Figure S11. NOX-1 single amino acid substitutions in NADPH-binding sites result in a complete lack of function. (**A**) Indicated strains were inoculated ($1X10^4$ conidia) and incubated for 3 days in darkness and 2 days in light at 30 °C. (**B**) $1X10^4$ conidia were inoculated in test tubes containing 3 mL of media and incubated as in **A** and conidia harvested and counted. Average of three independent experiments each with triplicates for each strain. (**C**) $1X10^3$ conidia were inoculated on solid media, incubated at 30 °C in the dark for 24 h, and radial growth of the colonies was measured (**D**). Average of three independent experiments each with triplicates for each strain. (**E**) Indicated strains were induced to develop protoperithecia as in Figure **S2**. (**F**) Conidia from indicated strains were induced to develop CATs and observed using confocal microscopy.
