# Supplementary material for: Neurospora crassa NADPH Oxidase NOX-1 Is Localized in the Vacuolar System and the Plasma Membrane
Source: Front Microbiol. 2019 Aug 14;10:1825. doi: 10.3389/fmicb.2019.01825 (PMC6702951; doi:10.3389/fmicb.2019.01825)
Supplement: Supplementary file 5 [file Data_Sheet_3.PDF]

Table S1. ***Neurospora crassa*** **strains used in this study**

Strain	Genotype	Source or reference
**Wild-type 74**	OR231 *mat A*	FGSC#987
**Wild-type 4200**	ORS-SL6 *mat a*	FGSC#4200
**FGSC-9717**	*Δmus-51::bar⁺; mat A his-3*	FGSC#9717
**FGSC-9718**	*Δmus-51::bar⁺; mat a*	FGSC#9718
Δnox-1 H10.1	*Δnox-1::hph⁺ mat a*	Cano-Domínguez et al., 2008
Δnox-1 H2.22	*Δnox-1::hph⁺ mat a his-3*	Cano-Domínguez et al., 2008
Δnox-1 his-3	*Δnox-1::hph⁺; mat a his-3*	This study. Progeny from *Δnox-1 H2.22* X 9717
Pccg-1-nox-1-mCherry Δnox-1 **d**	*Δnox-1::hph⁺; Δmus-51::bar⁺; his-3⁺::Pccg-1-nox-1::mcherry mat a*	This study. *Δnox-1 his-3* transformed with pNCNOX-1::mCherry 4
Pccg-1-nox-1-mCherry Δnox-1 **e**	*Δnox-1::hph⁺; Δmus-51::bar⁺; his-3⁺::Pccg-1-nox-1::mcherry mat a*	This study. *Δnox-1 his-3* transformed with pNCNOX-1::mCherry4
NOX-1P382H-mCherry Δnox-1	*Δnox-1::hph⁺; Δmus-51::bar⁺; his-3⁺::Pccg-1-nox-1^{P382H}::mcherry mat a*	This study. *Δnox-1 his-3* transformed with pNCNOX-1P382H::mCherry2
NOX-1C524R::mCherry Δnox-1	*Δnox-1::hph+; Δmus-51::bar⁺; his-3⁺::Pccg-1-nox-1^{C524R}::mcherry mat a*	This study. *Δnox-1 his-3* transformed with pNCNOX-1C524R::mCherry17
Pnox-1-nox-1::mCherry Δnox-1 20	*Δnox-1::hph⁺; Δmus-51::bar⁺; his-3⁺::Pccg-1-nox-1::mcherry mat a*	This study. *Δnox-1 his-3* transformed with pNCProm Nat NOX-1::mCherry4
Pnox-1-nox-1::mCherry Δnox-1 22	*Δnox-1::hph⁺; Δmus-51::bar⁺; his-3⁺::Pccg-1-nox-1::mcherry mat a*	This study. *Δnox-1 his-3* transformed with pNCProm Nat NOX-1::mCherry4
**VMA-1::GFP**	*his-3⁺::Pccg-1::vma-1::gfp mat A*	Bowman et al., 2015
**NCA-1::GFP**	*his-3⁺::Pccg-1::vma-1::gfp mat a*	Bowman et al., 2009
**ER7**	*Pgapdh::BIP::gfp::Phleo mat a.*	This study. Wild-type 4200 transformed with pGFP-BIP.
NCoS-7-5	*Δmus-51::bar⁺; his-3⁺::Pccg-1::c-Gly::GFP::ypt-52; mat A*	Seidel et al., 2013
**Nc28nor-1**	*Δnor-1::hph⁺ Δmus-*	Cano-Domínguez et al.,

Strain	Genotype	Source or reference
	51::bar^{+} *mat a*	2008
nor-1-gfp Δnor-1-7	*Δnor-1::hph*$^{+}$*; Δmus-51::bar*$^{+}$*; his-3*$^{+}$*::Pccg-1-nor-1::gfp mat a*	This study. *Nc28nor-1* transformed with pNCNOR-1::gfp18
nor-1-gfp Δnor-1-8	*Δnor-1::hph*$^{+}$*; Δmus-51::bar*$^{+}$*; his-3*$^{+}$*::Pccg-1-nor-1::gfp mat a*	This study. *Nc28nor-1* transformed with pNCNOR-1::gfp18

Table S2. Plasmids used in this study

Plasmid	Characteristics	Reference
**pMF272**	*Pccg-1::gfp*	Freitag M et al., 2004
**pJV-15-2**	*Pccg-1::mcherryfp*	Verdin et al., 2009
**pNCNOX-1::mCherry4**	*Pccg-1::nox-1::mcherryfp*	This study
**pNCNOX-1P382H::mCherry2**	*Pccg-1::nox-*1^{P382H}*::mcherryfp*	This study
**pNCNOX-1C524R::mCherry17**	*Pccg-1::nox-*1^{C524R}*::mcherryfp*	This study
**pNCPromNatNOX-1::mCherry4**	P*nox-1::nox-1::mcherryfp*	This study
**pAM01**	*PgpdA::KDEL BiP::gfp*	Meizoso-Huesca and Peraza-Reyes Unpublished
**pNCNOR-1::GFP18**	*Pccg-1::nor-1::gfp*	This study

Table S3. Oligonucleotides used in this study

Oligonucleotides	Sequence 5 → 3
NOX-1XbaIF2	GCTCTAGAATGAGTCTCCTCGTACTGCTG
NOX-1PacIR2	CCTTAATTAACGCGAAAGTGTTCCTTCCAG
Nox-1P382HF	CGTCACCCACTGGGCCTCCATCCTCAAG
Nox-1P382HR	GCCCAGTGGGTGACGCCAATACCCG
Nox-1C524RF	GTGTATTTCCGCGGGCCTAGTGCTGCTGC
Nox-1C524R-R	GCCCGCGGAAATACACTCCGACCGTCGTCC
PNC-NOX-1mCherry PromNatF	CAACCAAATCTAGAATGAGTCTCC
PNC-NOX-1mCherry PromNatR	GCGGCCGCCACCGCGGTGG
NOX-1PROM NATF	CAACCAAATCTAGAATGAGTCTCC
PromNatR	CATTCTAGATTTGGTTGGTTGGCGACCGCTAGATCCC
NOR-1BAMHIUP	GTGGATCCATGTCGCTAAAACAGG
NOR-1PacILOW	TTAATTAATATCTCCTGGACCCAGACC
His-3F	GGCACACAAATGGAAAACGGGACTCG
His-3R	CTGGCGTCAATTTCGCCTCGTTCG
Hyper secuenciarF	GCAATCACATCTTCACTACTTCAAATC
Hyper secuenciarR	CGAGGTCGACGGTATCGATAAGC
